# Accumulation of mutations in genes associated with sexual reproduction contributed to the domestication of a vegetatively propagated staple crop, enset

**DOI:** 10.1038/s41438-020-00409-7

**Published:** 2020-11-01

**Authors:** Kiflu Gebramicael Tesfamicael, Endale Gebre, Timothy J. March, Beata Sznajder, Diane E. Mather, Carlos Marcelino Rodríguez López

**Affiliations:** 1 Environmental Epigenetics and Genetics Group, Department of Horticulture, College of Agriculture, Food and Environment, University of Kentucky, Lexington, KY 40546 USA; 2 Policy Study Institute, P.O. Box: 2479, Addis Ababa, Ethiopia; 3 School of Agriculture, Food & Wine, The University of Adelaide, Waite Campus, Glen Osmond, SA Australia

**Keywords:** Plant domestication, Natural variation in plants

## Abstract

Enset (*Ensete ventricosum* (Welw.) Cheesman) is a drought tolerant, vegetatively propagated crop that was domesticated in Ethiopia. It is a staple food for more than 20 million people in Ethiopia. Despite its current importance and immense potential, enset is among the most genetically understudied and underexploited food crops. We collected 230 enset wild and cultivated accessions across the main enset producing regions in Ethiopia and applied amplified fragment length polymorphism (AFLP) and genotype by sequencing (GBS) analyses to these accessions. Wild and cultivated accessions were clearly separated from each other, with 89 genes found to harbour SNPs that separated wild from cultivated accessions. Among these, 17 genes are thought to be involved in flower initiation and seed development. Among cultivated accessions, differentiation was mostly associated with geographical location and with proximity to wild populations. Our results indicate that vegetative propagation of elite clones has favoured capacity for vegetative growth at the expense of capacity for sexual reproduction. This is consistent with previous reports that cultivated enset tends to produce non-viable seeds and flowers less frequently than wild enset.

## Introduction

Plant domestication and breeding can alter and shrink genetic diversity^1^. In some crop species, this entails a shift from sexual to vegetative propagation^2^. Enset (*Ensete ventricosum* (Welw.) Cheesman) is a hapaxanth diploid (2*n* = 18) plant that belongs to the Musaceae family^3^. In the wild, enset propagates by seed^4^. The native distribution of wild enset encompasses the eastern coast Africa, from South Africa to Ethiopia, and extends west into the Congo^5^. In Ethiopia, which is considered to be the centre of origin of *E. ventricosum*, wild enset grows mainly along riversides and deep forest, extending into cultivated land and gardens in some regions^6^.

Despite its wide distribution, enset has been domesticated only in the Ethiopian highlands and it is now grown as a crop mainly in the southern and south-western parts of Ethiopia^7^. In these regions, cultivated enset is propagated vegetatively from suckers. Ethiopia maintains more than 600 accessions of cultivated enset via vegetative propagation^8^. Due to its importance for food security in Ethiopia, enset has been called “the tree against hunger”^9^. Enset is known for its high yield, drought tolerance, high shade potential, broad agro-ecological distribution and long storage capacity^10^. Despite these positive features, enset has received little research attention^5^ and its genetic diversity is under threat from diseases, such as bacterial wilt and from pressures associated with human population growth^7^.

Genetic analysis of intraspecific variation in enset has mainly relied upon data for ‘anonymous’ molecular markers, such as amplified fragment length polymorphisms (AFLP)^11^, random amplified polymorphic DNA (RAPD)^12^, inter simple sequence repeats (ISSR)^13^ and microsatellites (simple sequence repeats (SSR))^14^. Given that enset is vegetatively propagated, genetic divergence among cultivars may be minimal^15^ and could be difficult to detect using these marker types. The full spread of enset diversity and distribution in Ethiopia can be exploited when using an approach that enables to compare large fractions of the genome of individuals and encompass larger cultivation regions.

Here, we report the use of AFLP and genotyping by sequencing (GBS) to 230 enset accessions (192 cultivated and 38 wild) and 141 enset accessions (120 cultivated and 21 wild), respectively. Datasets collected using these methods were used to investigate population structure of cultivated and wild enset accessions and to identify signatures of selection and domestication within the enset genome. Understanding the population divergence of cultivated and wild enset and genetic base of enset domestication provides a valuable foundation for enset conservation, breeding and genetic improvement, and predicting how the species will respond to the predicted increase in environmental challenges due to global warming. To our knowledge, this is the first application of NGS to a large number of accessions of wild and cultivated enset collected from a large geographic area.

## Results

### AFLP analysis

Based on the analysis of presence/absence data for 111 AFLP amplicons with lengths ranging from 51 to 350 bp, the observed heterozygosity and Shannon’s Index were higher for cultivated accessions (0.193 ± 0.02 and 0.298 ± 0.029) than for wild accessions (0.186 ± 0.02 and 0.285 ± 0.029). However, the average genetic distance between cultivated accessions was lower (0.026 ± 0.002) than between wild accessions (0.047 ± 0.007). The average percentage of polymorphic peaks for cultivated and wild accessions were 45.75 ± 3.25% and 41.7 ± 6.18%, respectively.

**Fig. 1:**
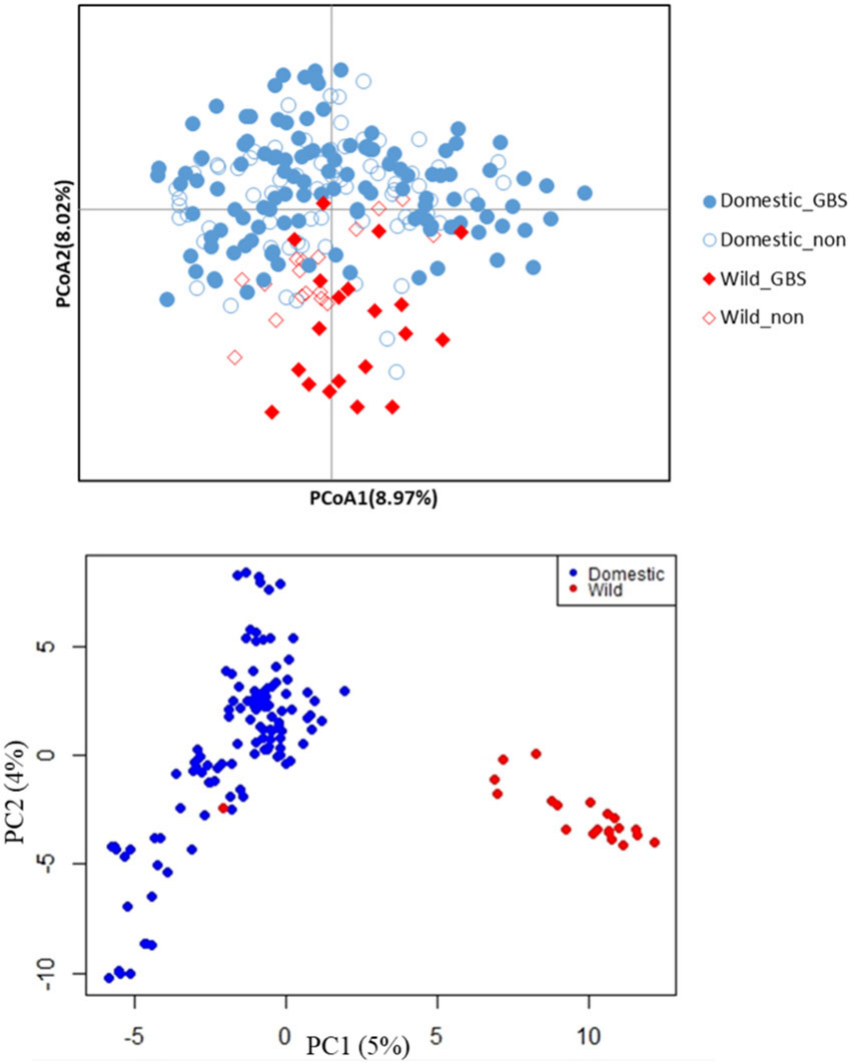
Genetic diversity of wild and cultivated enset in the six top enset producing regions of Ethiopia. **a** Principal coordinate analysis (PcoA) of AFLP markers genotyped in 192 cultivated and 38 wild enset samples. Solid symbols indicate 141 samples selected for GBS analysis, empty symbols indicate 89 accessions excluded from GBS analysis. **b** Principal component analysis (PCA) using 5169 GBS-based SNP markers generated from 120 cultivated and 21 wild enset accessions

Analysis of molecular variance (AMOVA) showed that the majority (87–89%) of enset genetic variability is explained by within-region differences, while 11–13% can be attributed to variation between regions (Table 1). Principal coordinate analysis (PCoA) using AFLP markers showed that wild and cultivated enset accessions formed clusters with considerable overlapping of individuals from the two groups (Fig. 1a).

**Table 1:** Analysis of molecular variance (AMOVA) using AFLP markers for 192 accessions of cultivated enset collected from six regions.

Source	df	SS	MS	Est.Var.	% of variation	*P*-value
Among regions	5	173.32	34.66	0.915	13	0.0001
Within regions	186	1175.02	6.32	6.317	87	
Total	191	1348.34	–	7.232	100	

*df* degrees of freedom, *Est.Var.* estimated variance, *SS* sum of squares, *MS* mean of sum of squares *%* percentage of variance explained.

### SNP discovery and analysis

GBS of 149 (125 cultivated and 24 wild) enset accessions generated a total of 569,324,179 reads with 74 bp length and 50% of GC content. Eight samples were removed because of high SNP missing ratio (>30%), leaving 141 samples (120 cultivated and 21 wild) for analysis.

A total of 3,743,487 tags passed mapping criteria when physically mapped to the *Musa acuminata* subsp. *malaccensis* (wild banana) genome. This reference genome-based SNP calling generated 22,884 SNPs showing locus coverage lower than 0.1 and minor allele frequency lower than 0.01. After filtering to remove SNPs with missing value >20% and missing ratio >30%, 5169 high-quality SNPs remained. Of these, 4282 SNPs (83%) physically mapped to one of the 11 chromosomes of the *M. acuminata* subsp. *malaccensis* genome and the remaining 887 SNPs were physically mapped to genome scaffolds (Supplementary Table 1). The number of SNPs per chromosome ranged from 251 in chromosome 2 to 465 in chromosome 4 (Supplementary Table 1), with an average 389 SNPs per chromosome. The highest density of SNPs was detected on chromosome 4 (65.05 kb/SNP) and the lowest on chromosome 10 (91.5 kb/SNP). A/G transitions presented the highest frequency (29.06%) followed by C/T transitions (28.03%) and A/C transversions (11.30%) (Supplementary Table 2).

### Genetic relatedness and population structure of cultivated and wild enset accessions

PCA using the 5169 SNP markers indicated that all but one of the wild enset accessions clustered separately from the cultivated enset accessions (Fig. 1b). We then performed the same analysis using the same number of wild and cultivated samples (21 per population) to ensure that the imbalance in population size would not skew the observed results. Samples from Dawro, Guragie, Sidama, and Omo (four from each), and five from Keffa were randomly selected, and performed PCA using their GBS data together with that from all 21 wild enset samples (Supplementary Fig. 1). Comparison of PCA plots showed that the percentage of variability explained by PCA was different for each data set (5% and 4% for all 141 samples and 10% and 4% for the reduced balanced samples set) Importantly, wild and domestic samples occupied similar relative positions across the eigenspace. The results of PCA analysis of the weighted and centred SNP marker data were consistent with Fig. 1b and Supplementary Fig. 1 confirming that wild and cultivated accessions form two separate clusters (Supplementary Fig. 2).

DAPC analysis showed a clear separation of enset accessions into three clusters (Supplementary Fig. 3a). Cluster 1 comprised 24 cultivated accessions and one wild accession. Among the 24 cultivated accessions in this clade, 17 accessions (71%) were collected from areas in which only cultivated enset was found. Cluster 2 contained 96 cultivated accessions, 67% of which were collected from areas which have both cultivated and wild enset accessions and the rest from cultivated only regions. Finally, all samples in Cluster 3 were wild accessions collected from regions containing domestic and wild accessions or from wild only regions. GenGIS display (Supplementary Fig. 3b) show consistent results with DAPC clustering.

Population structure analysis of cultivated and wild enset accessions was performed for both AFLP and SNP makers using STRUCTURE software. Individuals were considered part of a cluster when the probability of membership was 0.7 or greater. AFLP’s highest Δ*K* was at *K* = 5 followed by *K* = 3 (Supplementary Fig. 4). For *K* = 5, clusters 1, 2, and 4 included only cultivated accessions, cluster 5 was 81.5% cultivated accessions and cluster 3 was 87.5% wild accessions. For *K* = 3, clusters 1, 2, and 3 were 96% cultivated, 98% cultivated, and 86% wild enset accessions, respectively.

**Fig. 2:**
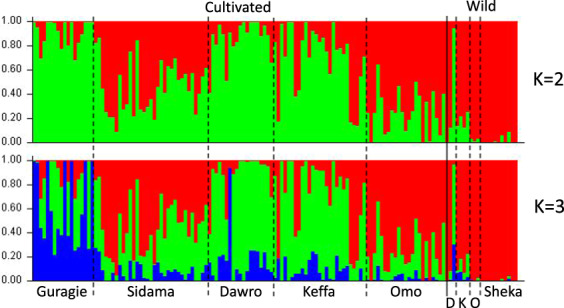
Estimated population structure of 141 cultivated and wild enset accessions analysed using the software STRUCTURE using 5169 GBS generated SNPs. Accessions are grouped first by cultivated or wild (separated by continuous vertical line) and then by their regions of origin (separated by dashed line). D Dawro, K Keffa, O Omo

Using SNPS markers, Δ*K* method indicated that the most informative number of subpopulations was two followed by three (Supplementary Fig. 4). In *K* = 2 (Fig. 2), cluster 1 comprises 50 individuals (20 wild and 30 cultivated accessions) and cluster 2 contains 57 individuals (one wild and 56 cultivated accessions). The pattern was similar at *K* = 3 membership patterns were similar to those observed for AFLP *K* = 3 STRUCTURE results, i.e. 31 cultivated accessions clustered with wild accessions in cluster 1, and clusters 2 and 3 harbours only cultivated accessions (Fig. 2). In cluster 2, 93% of the accessions were collected from areas where both cultivated and wild enset grows, in cluster 3 however, 86% of the accessions were collected from areas where only cultivated enset grows. Cultivated enset accessions showed lower membership values than wild accessions both for *K* = 2 and *K* = 3 (Supplementary Fig. 6).

When cultivated enset accessions were analysed on their own, the most informative number of subpopulations identified by Δ*K* analysis was 2 followed by 3. At *K* = 2, 54% of the accessions in cluster 1 and cluster 2 were from areas where cultivated and wild enset grow, the rest of the accession (46%) in both the clusters were collected from areas with only cultivated enset. All 24 accessions allocated to DAPC’s cluster 1 were assigned to STRUCTURE’s cluster 1 (20 of them with membership values >0.7 and the remaining 4 >0.6). In *K* = 3, all 13 accessions in cluster 3 and 53% of the accessions in cluster 2 were collected from areas where both cultivated and wild enset grows, and 57% of the accessions in cluster 1 were from areas where only cultivated enset grows (Supplementary Fig. 7).

### Genetic diversity of cultivated and wild enset

The average PIC and gene diversity were similar for cultivated and wild accessions. Cultivated enset accessions exhibited higher heterozygosity than wild accessions, while the average major allele frequency was higher for cultivated than for wild accessions (Table 2). The average genetic distances and average *F*_st_ values among cultivated and wild accessions were 0.33 ± 0.001 (SE) and 0.11 ± 0.005 (SE), respectively.

**Table 2:** Genetic diversity analysis of cultivated and wild enset accessions collected from six major enset producing regions of Ethiopia, analysed using all 5169 GBS-based SNP markers and 5011 neutral SNP markers.

SNPs used	Category	Sample size	Major allele frequency	Gene diversity	Heterozygosity	PIC	Inbreeding coefficient
All SNPs	Cultivated	120	0.71 ± 0.002	0.40 ± 0.003	0.26 ± 0.003	0.35 ± 0.003	0.34 ± 0.005
	Wild	21	0.68 ± 0.002	0.42 ± 0.002	0.21 ± 0.002	0.37 ± 0.002	0.52 ± 0.005
Neutral	Cultivated	120	0.71 ± 0.002	0.4 ± 0.003	0.27 ± 0.003	0.35 ± 0.002	0.33 ± 0.005
	Wild	21	0.67 ± 0.002	0.42 ± 0.002	0.22 ± 0.003	0.37 ± 0.002	0.51 ± 0.005

**Fig. 3:**
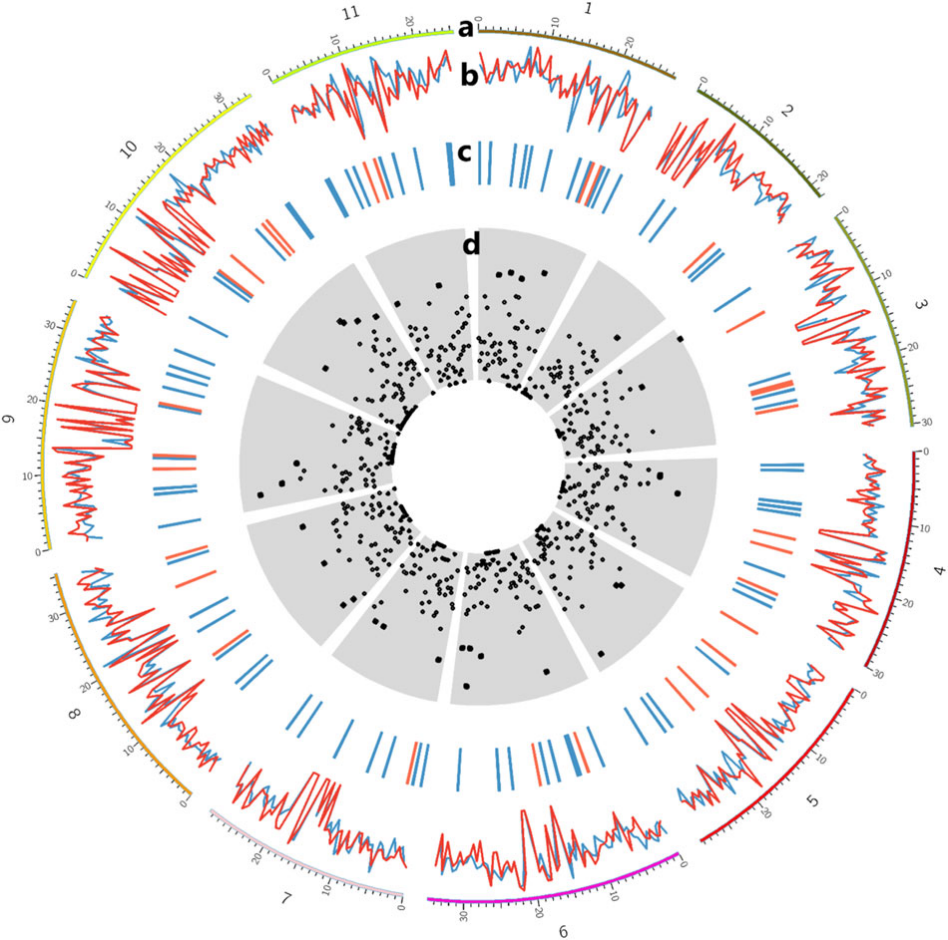
Summary of genetic diversity and genetic differentiation of cultivated and wild enset accessions measured within 500 kb sliding window drawn using circos plot. **a** *Musa acuminata* subsp. *Malaccensis* 11 Chromosomes portrayed along the perimeter of each circle (numbers indicate chromosome size in Megabases), **b** genetic diversity of cultivated (blue) and wild (red) enset accessions, genetic diversity for each sliding window was calculated nucleotide diversity divided by number of markers. **c** *F*_st_ < 0.02 (red) and >0.2 (blue), **d** total count of SNP markers per window, dots near the centre represent a low number of SNPs and the dots further out represent high numbers of SNPs

Analysis of genome-regional patterns of nucleotide diversity using 500 kb non-overlapping sliding windows showed that the average nucleotide diversity was higher in wild enset accessions (0.32 ± 0.005 (SE)) than in cultivated enset accessions (0.27 ± 0.006 (SE)) (Fig. 3). Calculation of the degree of diversification (*F*_st_) between cultivated and wild enset accessions identified a total of 29 genomic subregions with high degree of diversification (*F*_st_ > 0.2) and 76 genomic subregions with low *F*_st_ (*F*_st_ < 0.02) (Fig. 3). Chromosomes 3, 5, and 10 presented the highest number of genomic subregions with high *F*_st_. On the other hand, chromosome 1 presented the highest number of low *F*_st_ genomic subregions (11 subregions), while chromosomes 2, 3, and 5 showed the lowest number of low *F*_st_ genomic subregions (4 subregions).

### Linkage disequilibrium (LD)

The association between loci across genotypes was assessed to estimate the extent of genome-wide LD decay within cultivated and wild enset populations. The average LD between SNP markers within the 500 kb LD-window was low (*R*^2^ = 0.1 ± 0.014) with 5% of markers’ pairwise marker correlations presenting *R*^2^ ≥ 0.8. The average genome-wide LD (*R*^2^) for pairwise SNP combinations within 1, 10, and 100 kb was 0.4, 0.34, and 0.2, respectively. The proportion of SNP combinations that had high LD (*R*^2^ ≥ 0.8) was similar (5%) for both cultivated and wild enset populations. The overall average genome-wide LD for wild enset (*R*^2^ = 0.12 ± 0.0004) was slightly higher than for cultivated enset (0.10 ± 0.0013). However, wild enset had slower genome-wide LD decay (Supplementary Fig. 8). The maximum average genome-wide LD (*R*^2^) in wild population was 0.23 at 100 kb and declined to 0.08 at 200 kb physical distance, whereas in cultivated population the initial (maximum) average genome-wide LD was 0.21 at 100 kb and declined to 0.05 at 200 kb physical distance.

### Genomic regions under selection pressure

**Fig. 4:**
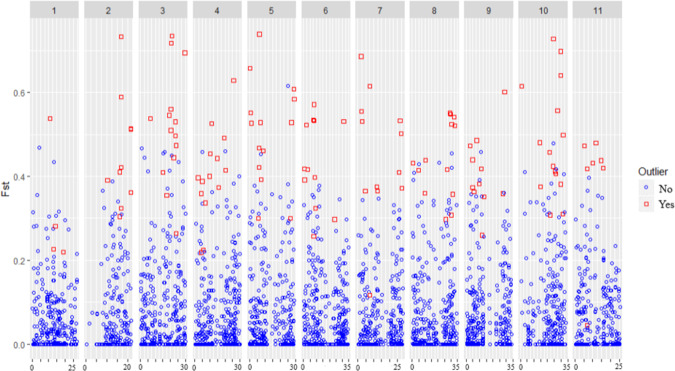
*Identification of genomic regions under selection pressure during enset domestication.* *F*_st_ values of 5169 SNP loci, displayed according to their genomic positions within 5Mb intervals on the 11 chromosomes of *Musa acuminata* subsp. *Malaccensis* genome

The genome scan approach (LOSITAN-based *F*_st_-outlier detection method) implemented in this study identified 158 (2.56%) SNPs, whose frequency was significantly different between cultivated and wild enset populations, which are dispersed throughout the 11 chromosomes of the wild banana reference genome (Fig. 4). Chromosomes 3 and 10 harbour highest number of outlier SNPs (16 outlier SNPs each). Chromosome 1 contains the lowest number of outlier SNPs (4 SNPs), despite containing the third highest number of SNP markers used for this analysis. Genetic diversity and population structure analysis for cultivated and wild enset accessions using 5011 neutral SNP markers (after removing outlier 158 SNP markers) was then performed. Observed results were consistent with the analysis performed using all 5169 SNP markers (Table 2 and Supplementary Fig. 8a).

Mapping of outlier SNPs to the reference genome (*M. acuminata* subsp. *malaccensis*) genome identified 89 genes containing one or more SNPs within their protein coding region (Supplementary Table 3). Of these, 18 genes (20%) were found to be associated to sexual reproduction traits, i.e. flowering (9 genes), seed development/germination (9 genes), and 2 (2%) have been previously associated to domestication in other species (Supplementary Table 4). The function of these genes was annotated based on comparative genomics (one gene), deduced from protein containing domains as putative function (five genes) and experimentally validated (14 genes) in other plants such as *Arabidopsis thaliana*, rice, soybean, and tomato. We then used a PCA-based outlier marker detection approach, *pcadapt*, to validate the SNPs identified using LOSITAN. In total, *pcadapt* analysis identified 373 outlier SNPs, of which, 51 were common for both approaches. Out of these, 24 outlier SNPs were found in 23 genes containing SNPs identified using Lositan (Supplementary Table 3). Of these, 4 (17%) were found to be associated to sexual reproduction traits, i.e. flowering (3 genes), seed development/germination (1 gene) (Supplementary Table 4).

PCA, DAPC, and STRUCTURE results generated using neutral SNP markers showed similar results to those obtained using the full set of markers (Supplementary Fig. 7 and Supplementary Tables 5 and 6). In brief, PCA showed the same clustering of wild and domestic samples with a similar percentage of variability explained by the first two PCs (9% and 8% for analysis performed with all SNPs and neutral SNPs only, respectively) (Supplementary Fig. 9a). All but 6 accessions were allocated to the same clusters by DAPC using both data sets. These 6 accessions switched between cluster 2 (for all SNPs) and 1 (for neutral SNPs only) (Supplementary Table 5). STRUCTURE analysis using neutral markers also identified *K* = 2 and *K* = 3 as the optimal number of accession clusters (Supplementary Fig. 9b, c). As observed in DAPC analysis, accession cluster allocation was very similar using both datasets. For *K* = 2 five cultivated accessions switched from cluster 1 to cluster 2. For *K* = 3 one cultivated accession switched from cluster 2 to cluster 1, four accessions that were assigned to cluster 2 using all SNPs were not clustered when using neutral markers and 2 accessions not clustered using all markers were assigned to cluster 1 when using neutral markers (Supplementary Table 6). The use of neutral SNPs did not have an effect on the cluster assignment of wild accessions.

## Discussion

### Genetic diversity of enset in Ethiopia

The results presented here indicate that cultivated and wild enset accessions exhibit similar gene diversity and polymorphic information content (PIC). This is similar to what has been reported based on SSR marker analysis of enset genetic diversity^14^, but differs from what has been reported by Olango et al.^16^, who reported higher gene diversity in cultivated (0.59) than in wild enset populations (0.40), but similar hetrozygosity levels (0.5). The genetic diversity for both cultivated and wild enset reported in the current study is lower than previous enset genetic diversity studies conducted using SSR makers^14,16^. Observed differences might be due to the nature of the different types of markers used. SSRs and microsatellite are multi-allelic and are more polymorphic than SNP markers, which are usually bi-allelic. The genetic diversity detected here for enset is higher than what has been reported for some other vegetatively propagated plants such as Cassava^17^, and out-crossing plants such as sunflower^18^ but lower than what has been reported for Japonica rice^19^.

Our observations that cultivated enset exhibit higher heterozygosity and Shannon’s Index than wild enset resemble what have been reported for enset based on SSR markers^14^ and for other plant species, including *Camellia sinensis*^20^ and *C. taliensis*^21^. The high heterozygosity of cultivated enset might be due to vegetative propagation maintaining heterozygosity across clonal generations. In addition, the wild enset habitat has been sharply declining in Ethiopia because of population growth and deforestation^16,22^, which is consistent with the high inbreeding coefficient in wild enset population observed in our results. This reduction in effective population size might have contributed to the observed lower heterozygosity due to the increase of chances of inbreeding in wild enset populations. Interestingly, contrary to what have been reported for other vegetative propagated crops^23^ our LD analysis showed a slower LD breakdown in wild than in cultivated enset. Models on the relation between LD and inbreeding indicate that higher levels of inbreeding and smaller effective population size lead to lower LDs^24^. Whether the observed differences in LD and inbreeding observed here are real or associated to the imbalance in tested population sizes needs to be determined with further studies. Genetic distances were greater among wild accessions than among cultivated accessions, possibly because wild populations remained isolated by distance or geographical barriers^13^, while cultivated materials were more readily transferred between regions through regular long-distance accessions exchange between farmers^25^ combined with rare sexual reproduction events^4^. Limited genetic distances among cultivated enset accessions could also be due to recent separation (fragmentation) of the varieties, without sufficient evolutionary time to generate variation^26^.

### Population structure and genetic relationship between cultivated and wild enset accessions in Ethiopia

PCA revealed that cultivated and wild enset accessions separated into genetically distinct clusters despite being morphologically similar members of the same taxonomic species. PC1 separated wild and cultivated clusters, while PC2 captured the variability between cultivated samples. It is possible that cultivated enset and the current wild enset in Ethiopia originated from different ancestral materials. Interestingly, the percentage of the total variability explained by each PC when using a balanced number of samples was very similar (5% and 4% for PC1 and 2, respectively) indicating that both clusters are not so dissimilar.

DAPC analysis indicate that cultivated and wild enset group into two and one clusters, respectively. On the contrary, bayesian STRUCTURE analysis suggest that two clusters is the optimum number of groups. However, when STRUCTURE analysis was performed for cultivated accessions only, the optimum number of clusters was two, supporting DAPC results. and this is similar to previous SSR marker-based enset genetic diversity study^16^. Analysis of the membership probability values show that wild accessions tend to be “pure wild” (membership probability >0.9). cultivated enset accessions clustering was poorer with up to ***~***30% of the accessions presenting membership probabilities below 0.7 suggesting higher levels of admixture. Some cultivated accessions clustered with wild accessions, possibly indicating recent introgression of wild enset into farming systems^24^. In the Omo region, particularly in the Ari sub-region, wild enset growing in gardens have been adopted by farmers as a cultivated crop and propagated^27^. Thus, multiple domestication events and/or frequent introgression from wild enset could explain the high genetic diversity and overlapping spatial distributions of wild and cultivated enset^5^. These results coincide with recent findings indicating that although cultivated enset has been traditionally thought to be propagated vegetatively, it may, sporadically, sexually cross with wild accession^4^.

### Loci under selection signature

Improved understanding of the genetic adaption of enset could facilitate genetic improvement. *F*_st_ outlier tests for detecting extreme allele frequency differentiation can detect genomic regions that have evolved under adaptation and selection^28^. Here, combined lositan and R package *pcadapt* identified 51 *F*_st_ outlier SNP markers that show significant (*P* < 0.01) genetic differentiation between cultivated and wild enset. Mapping of these outlier markers to the diploid banana genome led to the identification of 23 genes under selection during enset domestication. 17% of these genes were found to be related to the regulation of flowering, or seed development.

Certainly, it has been suggested that vegetative reproduction during domestication can lead to the loss of sexual reproductive capacity^2^, and that flowering and seed development are important characteristics that differentiate cultivated and wild enset^5^. Wild enset flowers more frequently has larger flowers (mean basal girth 186 cm) than cultivated enset (mean basal girth 106 cm)^29^. Wild enset is highly prolific, producing thousands of large (about 12 mm diameter) hard black seeds, while cultivated enset plants bear fewer seeds, which are small (3 mm), soft, pale, and incompletely developed^29^. It has been previously suggested that these traits could be due to reduced fitness resulting from a subsequent selection and domestication bottleneck^30^. The proportion of genes found to be under selection in cultivated enset, indicate that selection associated to domestication could be the driver of those traits. However, a recent study reported that although seeds from wild and domestic enset present significant differences in weight and germination behaviour, no differences were observed in seed viability, time to germination, and internal morphology^4^. Further larger and targeted studies are needed to determine if such evidence on differences in seed biology between wild and domestic enset are associated to the genetic variability described here.

In addition, the calculation of degree of diversification (*F*_st_) between cultivated and wild enset accessions enabled the identification of genomic subregions (500 kb non-overlapping) with high (*F*_st_ > 0.2) degree of diversification. Genomic subregions with high *F*_st_ may contain or be associated to potential genes that are related to plant domestication and adaptions. In the current study, *F*_st_ outlier-based scan for candidate genes under putative selection and adaptation has found promising results, and is an important step forward to further studies on gene mapping and identification, and designing enset breeding programme.

## Materials and methods

### Study area

Samples were collected from six of the major enset producing regions in Ethiopia: Dawro, Guragie, Keffa, South Omo, Sheka, and Sidama (Supplementary Fig. 10). Within each of these regions, samples were collected from subregions and from two or three districts within each subregion (Supplementary Table 7). Within each subregion, samples of domesticated enset were collected from five to ten households, selected based on recommendations from local agricultural extension experts. Samples of wild enset were collected around farming areas, along riversides, and in deep forests. For each sampling location, latitude and longitude (degrees, minutes, seconds) were collected using GPS essentials mobile app (https://downloads.tomsguide.com/GPS-Essentials,0301-49666.html) and then transformed to standard Universal Tranverse Mercator coordinates (UTM) using a geographic unit converter (http://www.rcn.montana.edu/Resources/Converter.aspx) (Supplementary Table 7).

### Sample collection and DNA extraction

Leaf samples were collected from 230 (192 cultivated and 38 wild) enset plants. Accessions collected from farms were between 1 and 2 years old (based on the farmer’s information). Such accessions were grown as part of a plantation in a large field or within household gardens as ornamental or as animal feed. At the time of sampling, farmers were requested to describe every accession as wild or cultivated (Supplementary Table 8). Each sample consisted of a 5 cm × 5 cm fragments of the leaf blade of the most recently unfurled leaf. Each sample was placed in a 50 ml tube and stored on ice during transportation, then stored at −80 °C until DNA extraction. Each subsample (80–90 mg) were milled using a mortar and pestle immersed in liquid nitrogen. DNA extractions were performed using DNeasy Plant Mini Kits (Qiagene Inc.) according to the manufacturer’s instructions. DNA concentration was measured using the QuantiFluor^(R)^dsDNA System^(a)^ (Promega, USA) following manufacturer’s instructions, then adjusted to 20 ng/µl using molecular biology grade water (Sigma).

### AFLP preparation and analysis

AFLP reactions^31^ were performed for all 230 samples using a modification of the protocol described by López et al.^32^. Briefly, samples containing 55 ng of genomic DNA were enzymatically digested in a 12.5 reaction volume containing *Mse*I, *EcoR*I (NEB) and ligated to *Mse*I and *EcoR*I adaptors (Supplementary Table 9) at 37 °C for 2 h in a T100^TM^ Thermal cycler (*Bio-Rad* Laboratories, Hercules, CA). Success of the digestion/ligation reaction was confirmed on 1.5% of agarose gel electrophoresis. Pre-selective PCR amplification was carried out using primers containing a 3′ selective nucleotide (i.e., *EcoR*I = A and *Mse*I = C). Selective amplification was then conducted using a primer combination with three selective nucleotides at the 3′ ends (*EcoR*I = *ACG*) and *Mse*I = *CAA*). Selective bases were chosen according to previous work on enset^11^. PCR products were separated using Applied Biosystems 3130/3130xl Genetic Analysers (Applied Biosystems Life Technologies).

AFLP profiles were analysed using GeneMapper^®^ Software v4.0. Clear and unambiguous polymorphisms were considered and were scored on a presence/absence basis for each marker. Clearly polymorphic peaks were verified manually and scored as present (1) or absent (0) for each sample. The level of AFLP polymorphism and genetic diversity across enset accessions were examined using GenAlEx 6.502^33^ based on average band frequency, Nei’s unbiased genetic distance, PCoA, and AMOVA.

### GBS preparation and analysis

Genotyping-by-sequencing was conducted for 149 enset samples (125 domestic and 24 wild; Supplementary Table 10) that were selected to capture the genetic diversity shown by AFLP. The GBS library preparation was carried out as described by Xie et al.^34^ including a water negative control as described by Konate et al.^35^. The DNA concentration of each individual library was normalized to 5 ng/µl. Two pooled libraries were created, each by pooling the individual libraries from 75 uniquely barcoded samples (25 ng per sample) (Supplementary Table 9). Each pooled library was then amplified in 10 PCR reactions, each containing 10 µl of digested/ligated DNA library, 12.5 µl of NEB MasterMix, 2 µl of 10 µM forward and reverse Illumina_PE primers (Supplementary Table 8) and 0.5 µl of molecular biology grade water (Sigma). The amplification reaction was carried using a T1000 Thermocycler at 95 °C for 30 s, 16 cycles of (95 °C for 30 s, 62 °C for 20 s, 68 °C for 30 s) and 72 °C for 5 min. Amplification products were pooled together and cleaned using AMPure XP beads (Beckman Coulter, Australia) (1:1 ratio) to remove excess primers and unremoved adaptors. Libraries were sequenced using an Illumina NextSeq High Output 75 bp single-end run (Illumina 1.9 Inc., San Diego, CA, United States) at the Australian Genome Research Facility (AGRF, Adelaide, SA, Australia).

### GBS SNP calling

SNP calling was performed using two pipelines: de novo-based (reference genome independent) TASSEL-UNEAK pipeline^36^ and the reference-based TASSEL-GBS pipeline^37^. Only sequences containing identical matches to the barcodes followed by the expected sequence of three nucleotides remaining from a *Msp*I cut-site (5′-CGG-3′) were selected for the identification of SNPs. FASTQ files containing barcoded sequence reads were demultiplexed using unique barcodes for each sample and trimmed to 64 bp (not including the barcodes). Identical sequence reads were collapsed into tags and sequencing tags from the four NextSeq Illumina sequencing lanes were merged to form one master tag. Reads with Minimum kmer count (number of reads) <10 and Kmer Length <20 were removed from downstream analysis. These sequence tags were mapped to the wild (diploid) banana (*M. acuminata* ssp. *malaccensis*) genome sequence^38^ to deduce their genomic position using default parameters. Tags with single base pair mismatches between samples were considered as SNPs and were generated in Hapmap format. SNPs were further filtered for 1% of minimum minor allele frequency (MAF) and 70% of minimum locus coverage (mnLCov). Only SNPs that were generated via the reference-based SNP calling were used for downstream analysis and SNP generated by de novo-based were used for preliminary analysis, i.e AFLP-weighted PCA of the SNP data.

### Genetic diversity and population structure analysis

Genetic diversity and genetic differentiation (*F*_st_) were calculated using PopGenome R package^39^. Heterozygosity (the proportion of heterozygous individuals in the population), gene diversity (expected heterozygosity), PIC and inbreeding coefficient were calculated using Power Marker V3.25^40^. To examine the relationship between cultivated and wild enset accessions, PCA was built using TASSEL 5^41^. GenGIS^42^ was used to display the phylogenetic tree with the geographic regions of sample collection.

To confirm that the accessions submitted to genotyping-by-sequencing represented genetic variation as detected based on AFLP data, we additionally performed AFLP-weighted PCA of the SNP data. In particular, for each accession, using a grid defined by the first and second principal components of the PCA of AFLP marker data, we calculate weights defined as a ratio of the total number of accessions in a grid cell to the number of accessions submitted to genotyping-by-sequencing within this grid cell. Therefore, for each accession submitted to genotyping-by-sequencing, the weights reflected how many data points an accession represented in the space defined by PCA of the AFLP data.

Population structure was analysed using descriptive analysis of principal components (DAPC)^43^ and STRUCTURE^44^. The software STRUCTURE was used to analyse the hierarchical population structure by setting the length of the burn-in period to 50,000 iterations and number of the MCMC replications after burn-in to 50,000. Between two to nine population clusters (*K*) were considered, with 10 iterations conducted for each *K*-value. The best *K*-value was determined using structure Harvester based on delta *K*(Δ*K*) and maximum log likelihood L(*K*). The association between two alleles at two loci was assessed to investigate the genome-wide LD decay within cultivated and cultivated enset accessions. LD among the intra-chromosome SNP markers was estimated using Plink software^45^. The LD between pair of markers was calculated as the squared allele frequency correlation (*R*^2^) between pairs of SNP markers within chromosomes. The average genome-wide LD was plotted against the genetic distance (kb) between the SNP markers.

Genome-wide nucleotide diversity (average pairwise nucleotide differences) and population differentiation (*F*_st_) within and between wild and cultivated populations were calculated using a 500 kb non-overlapping sliding window. To obtain genetic diversity per window, nucleotide diversity was divided by number of SNPs per sliding window. These statistics were calculated using R package PopGenome^39^ and plotted using Circos^46^ to visualize the pattern of genetic diversity across the whole enset genome.

To detect loci under selection during enset domestication and adaptation, the FDIST2 method adopted by Beaumont and Nichols^47^ applied using lositan software^48^*. F*_st_ value was calculated for each SNP using allele frequencies conditional on expected heterozygosity (*H*_e_), and *P-*values for each SNP were calculated. SNPs within tags assigned to one of the wild banana chromosomes were used to identify *F*_st_ outliers. *F*_st_ outlier analysis was carried out with 50,000 interactions at 99% confidence interval. Then we searched for genes containing these outlier SNPs in the wild banana genome to identify potential genes under selection during enset domestication using magrittr R package^49^ and generated gene ID. The putative function of these genes was searched using UNIPROT database (https://www.uniprot.org/) based on the gene ID. Identified outliers were validated using R package *pcadapt*^50^. The outlier marker detection using *pcadapt* was performed based on the performed *pcadapt* analysis based on Benjamini–Hochberg procedure and outlier SNPs with >99% significant (<1% *p*-value) were considered.

Finally, PCA, DAPC, and STRUCTURE analyses were repeated using a data set of 5011 neutral SNP markers (i.e. after removing all SNPs under selection identified using Lositan).

## Supplementary information

Supplementary Fig.1

Supplementary Fig.2

Supplementary Fig.3

Supplementary Fig.4

Supplementary Fig.5

Supplementary Fig.6

Supplementary Fig.7

Supplementary Fig.8

Supplementary Fig.9

Supplementary Fig.10

Supplementary Table 1

Supplementary Table 2

Supplementary Table 3

Supplementary Table 4

Supplementary Table 5

Supplementary Table 6

Supplementary Table 7

Supplementary Table 8

Supplementary Table 9

## Data Availability

Sequencing and metadata is available on SRA database under the accession number PRJNA625659.
