# Supplementary material for: Accumulation of mutations in genes associated with sexual reproduction contributed to the domestication of a vegetatively propagated staple crop, enset
Source: Hortic Res. 2020 Nov 1;7:185. doi: 10.1038/s41438-020-00409-7 (PMC7603512; doi:10.1038/s41438-020-00409-7)
Supplement: Supplementary file 1 — Supplementary Fig.1 [file 41438_2020_409_MOESM1_ESM.pdf]

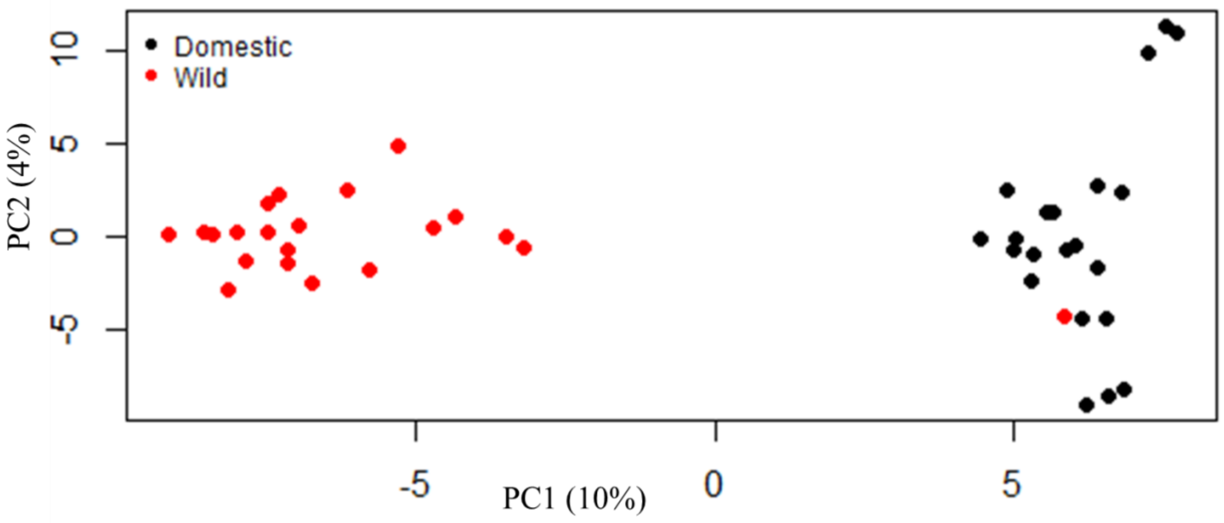

Supplementary Fig.1 PCA of 21 cultivated accessions randomly selected from the five sample collection regions (Guragie (4), Sidama (4), Omo (4), and Keffa (5) regions) and 21 wild enset accessions using 5169 SNP marker.
