# Supplementary material for: Accumulation of mutations in genes associated with sexual reproduction contributed to the domestication of a vegetatively propagated staple crop, enset
Source: Hortic Res. 2020 Nov 1;7:185. doi: 10.1038/s41438-020-00409-7 (PMC7603512; doi:10.1038/s41438-020-00409-7)
Supplement: Supplementary file 2 — Supplementary Fig.2 [file 41438_2020_409_MOESM2_ESM.pdf]

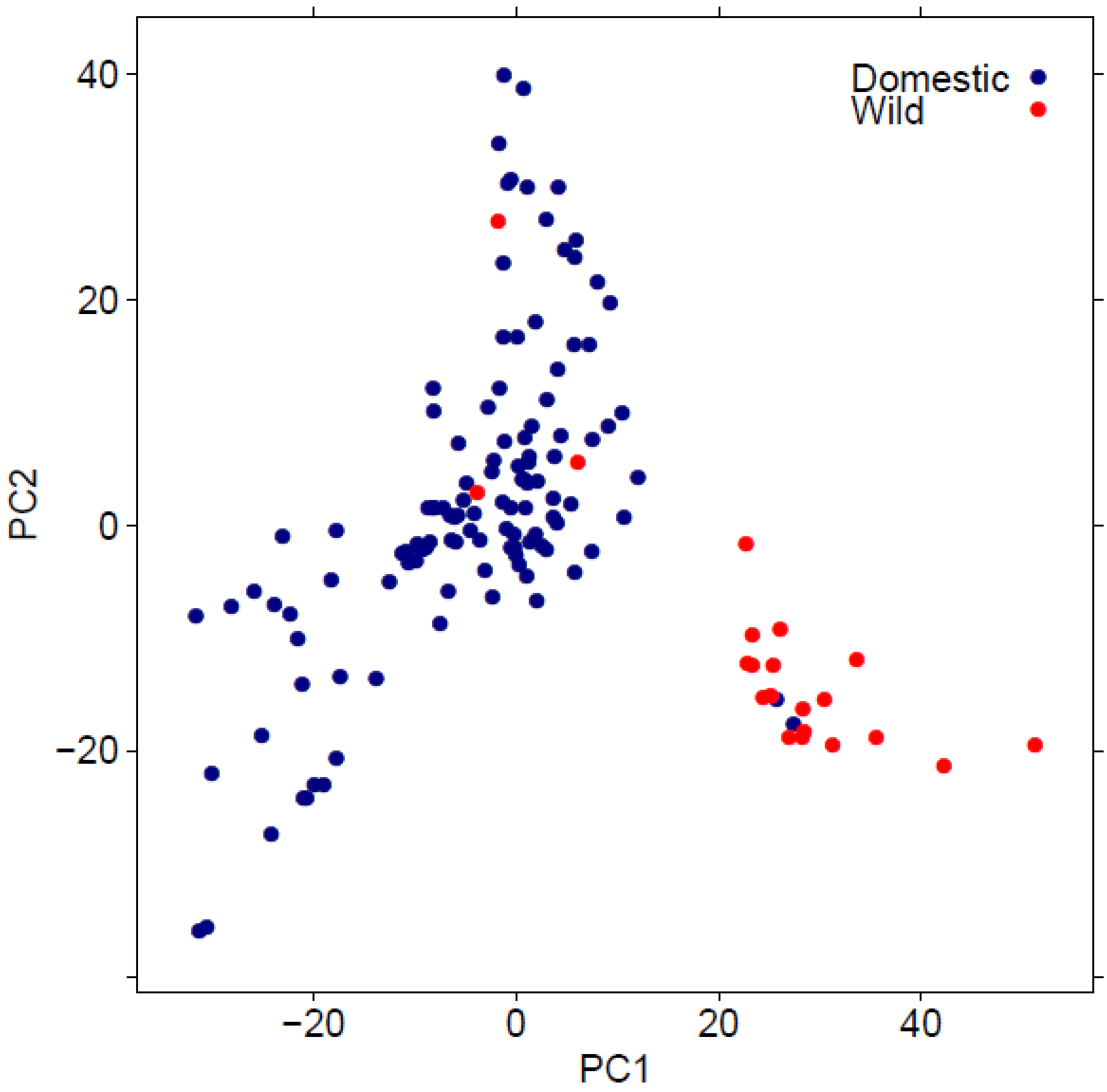

Supplementary Fig.2. Weighted PCA of cultivated and wild enset accessions using 4143 SNP markers generated using denovo-SNP (without reference genome) calling. The PCA generated using these SNP were weighted with 87 AFLP markers.
