# Supplementary material for: Accumulation of mutations in genes associated with sexual reproduction contributed to the domestication of a vegetatively propagated staple crop, enset
Source: Hortic Res. 2020 Nov 1;7:185. doi: 10.1038/s41438-020-00409-7 (PMC7603512; doi:10.1038/s41438-020-00409-7)
Supplement: Supplementary file 3 — Supplementary Fig.3 [file 41438_2020_409_MOESM3_ESM.pdf]

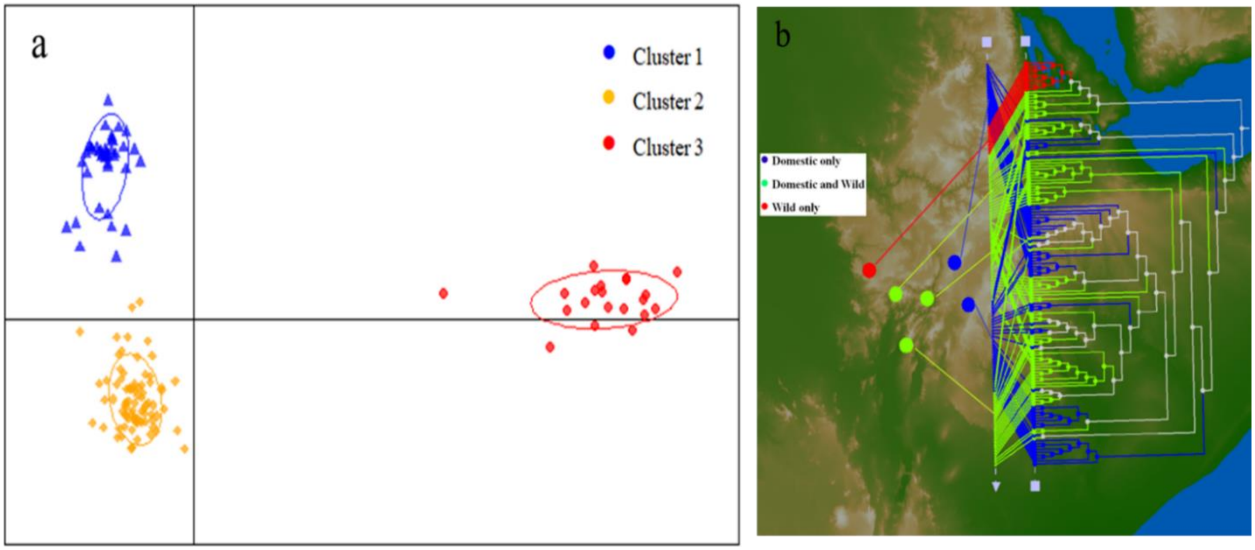

Supplementary Fig.3 Genetic structure of 141 enset accessions using 5169 genome-wide SNP markers **a**) population genetic structure using Discriminant Analysis of Principal Components (DAPC), **b)** GenGIS plot for the three clusters plotted with phylogenetic tree combined with the corresponding regions of collection. Samples were collected from different regions, regions with both domestic and wild, only domestic and only wild enset accessions.
