# Supplementary material for: Accumulation of mutations in genes associated with sexual reproduction contributed to the domestication of a vegetatively propagated staple crop, enset
Source: Hortic Res. 2020 Nov 1;7:185. doi: 10.1038/s41438-020-00409-7 (PMC7603512; doi:10.1038/s41438-020-00409-7)
Supplement: Supplementary file 4 — Supplementary Fig.4 [file 41438_2020_409_MOESM4_ESM.pdf]

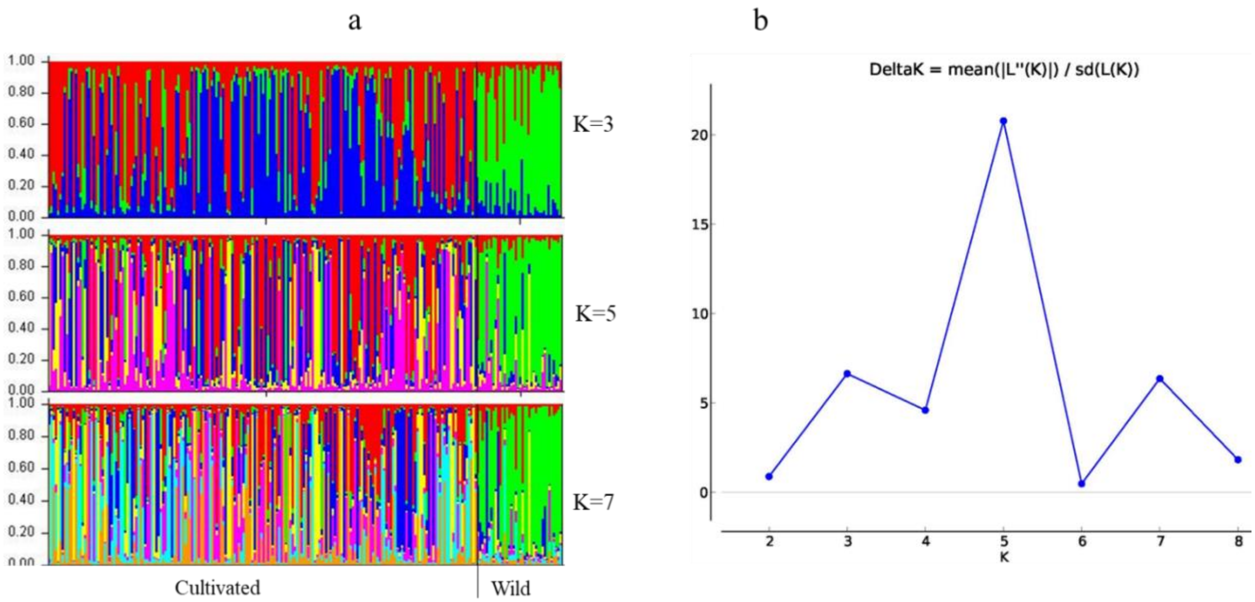

Supplementary Fig.4. a) Population structure analysis of wild and cultivated enset accessions using AFLP markers for K=5, 3and 7. Accessions are separated by cultivated (left) and wild (right). b) evanno plot of Delta K calculated from K ranging from 2 to 9 analyzed using Structure-Harvester.
