# Supplementary material for: Accumulation of mutations in genes associated with sexual reproduction contributed to the domestication of a vegetatively propagated staple crop, enset
Source: Hortic Res. 2020 Nov 1;7:185. doi: 10.1038/s41438-020-00409-7 (PMC7603512; doi:10.1038/s41438-020-00409-7)
Supplement: Supplementary file 5 — Supplementary Fig.5 [file 41438_2020_409_MOESM5_ESM.pdf]

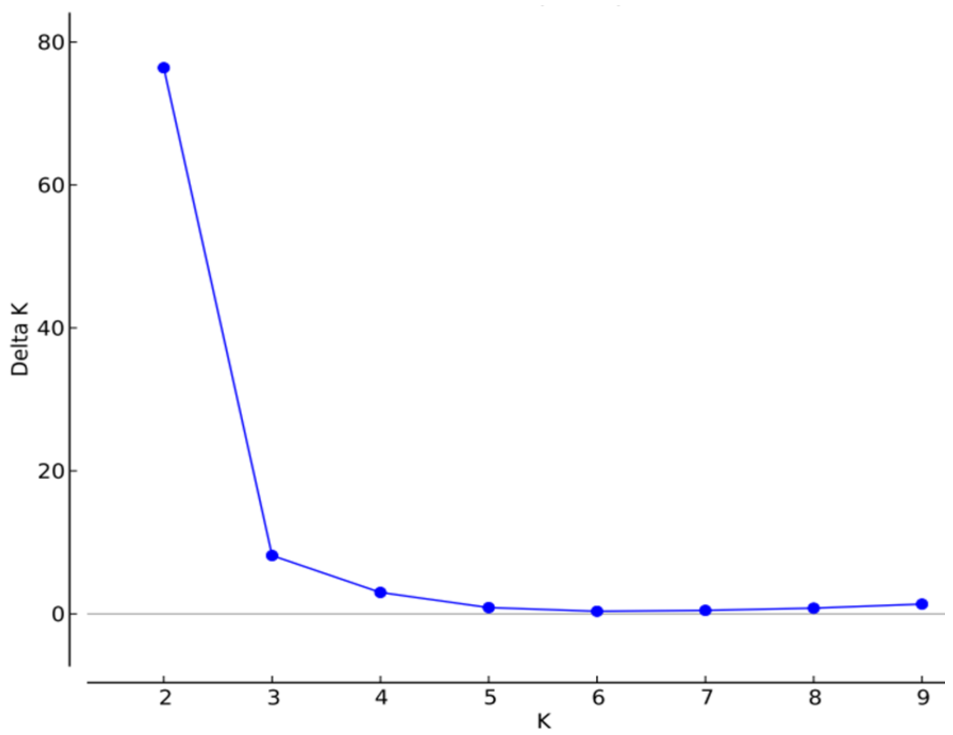

Supplementary Fig.5. Evanno plot of *Delta K* calculated from K ranging from 2 to 9 (each K repeated 10 times) for 5169 GBS generated SNPs from 141 cultivated and wild enset accessions analysed using Structure-Harvester.
