# Supplementary material for: Accumulation of mutations in genes associated with sexual reproduction contributed to the domestication of a vegetatively propagated staple crop, enset
Source: Hortic Res. 2020 Nov 1;7:185. doi: 10.1038/s41438-020-00409-7 (PMC7603512; doi:10.1038/s41438-020-00409-7)
Supplement: Supplementary file 6 — Supplementary Fig.6 [file 41438_2020_409_MOESM6_ESM.pdf]

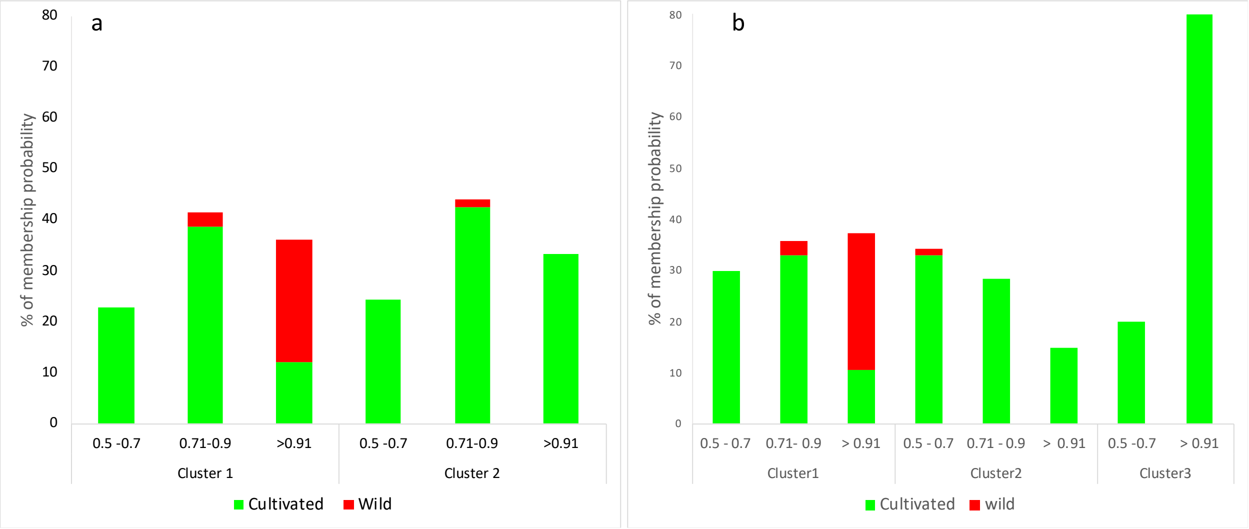

Supplementary Fig.6. Proportion of wild (red) and cultivated (green) enset accessions grouped according to their probability of membership to STRUCTURE defined clusters for K=2 (a) and K=3.
