# Supplementary material for: Accumulation of mutations in genes associated with sexual reproduction contributed to the domestication of a vegetatively propagated staple crop, enset
Source: Hortic Res. 2020 Nov 1;7:185. doi: 10.1038/s41438-020-00409-7 (PMC7603512; doi:10.1038/s41438-020-00409-7)
Supplement: Supplementary file 7 — Supplementary Fig.7 [file 41438_2020_409_MOESM7_ESM.pdf]

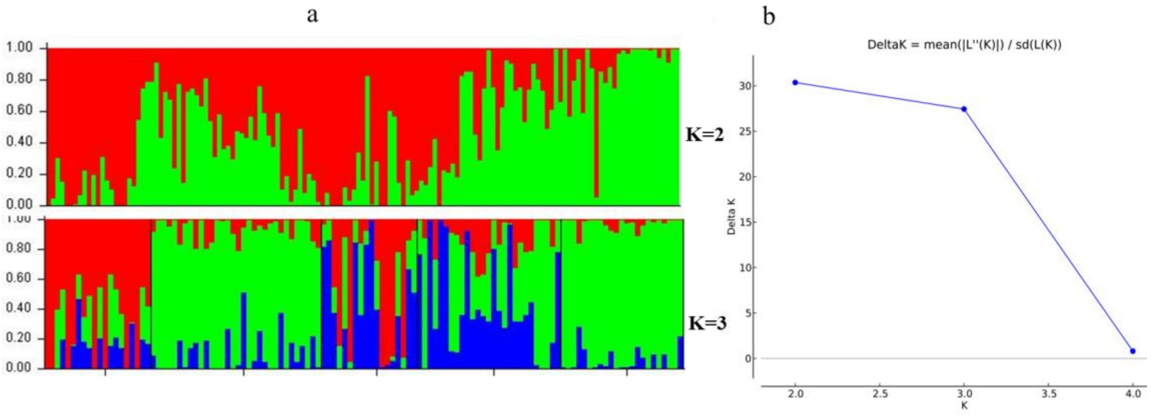

Supplementary Fig.7. a) population structure analysis of 121 cultivated enset accessions using 5169 SNP markers, plots indicating for K=2 and 3. b) Evanno plot of Delta K calculated from K ranging from 2 to 5 analyzed using Structure-Harvester.
