# Supplementary material for: Accumulation of mutations in genes associated with sexual reproduction contributed to the domestication of a vegetatively propagated staple crop, enset
Source: Hortic Res. 2020 Nov 1;7:185. doi: 10.1038/s41438-020-00409-7 (PMC7603512; doi:10.1038/s41438-020-00409-7)
Supplement: Supplementary file 8 — Supplementary Fig.8 [file 41438_2020_409_MOESM8_ESM.pdf]

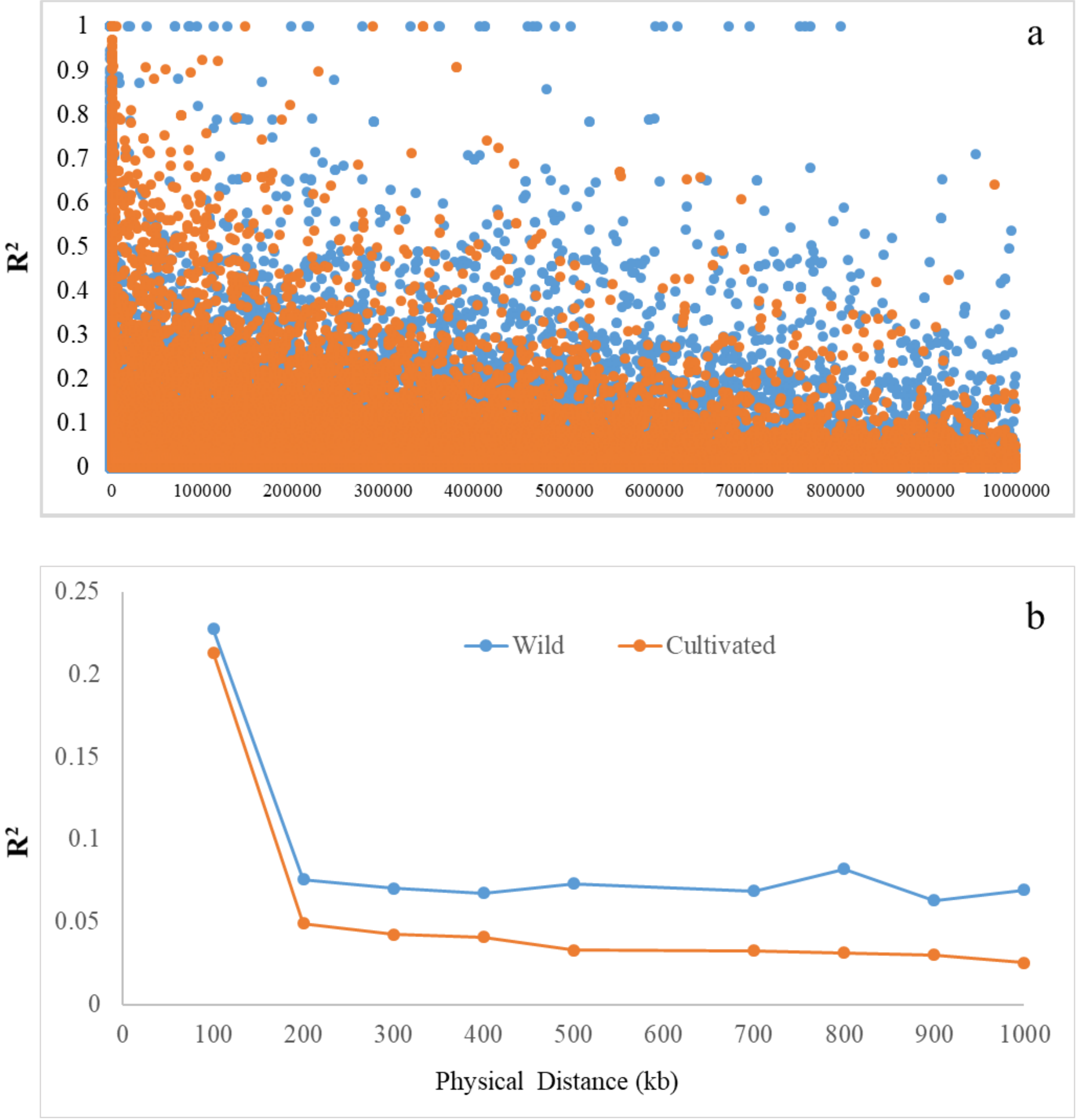

Supplementary Fig.8. a) genome-wide LD plot for cultivated (orange) and wild (blue) enset accessions. The squared allele frequency correlations (R2) between pair of intra-chromosomal SNPs were plotted against the physical distance between these markers, b) genome-wide linkage disequilibrium decay (R2) of cultivated (orange) and wild (blue) enset population.
