# Supplementary material for: Accumulation of mutations in genes associated with sexual reproduction contributed to the domestication of a vegetatively propagated staple crop, enset
Source: Hortic Res. 2020 Nov 1;7:185. doi: 10.1038/s41438-020-00409-7 (PMC7603512; doi:10.1038/s41438-020-00409-7)
Supplement: Supplementary file 9 — Supplementary Fig.9 [file 41438_2020_409_MOESM9_ESM.pdf]

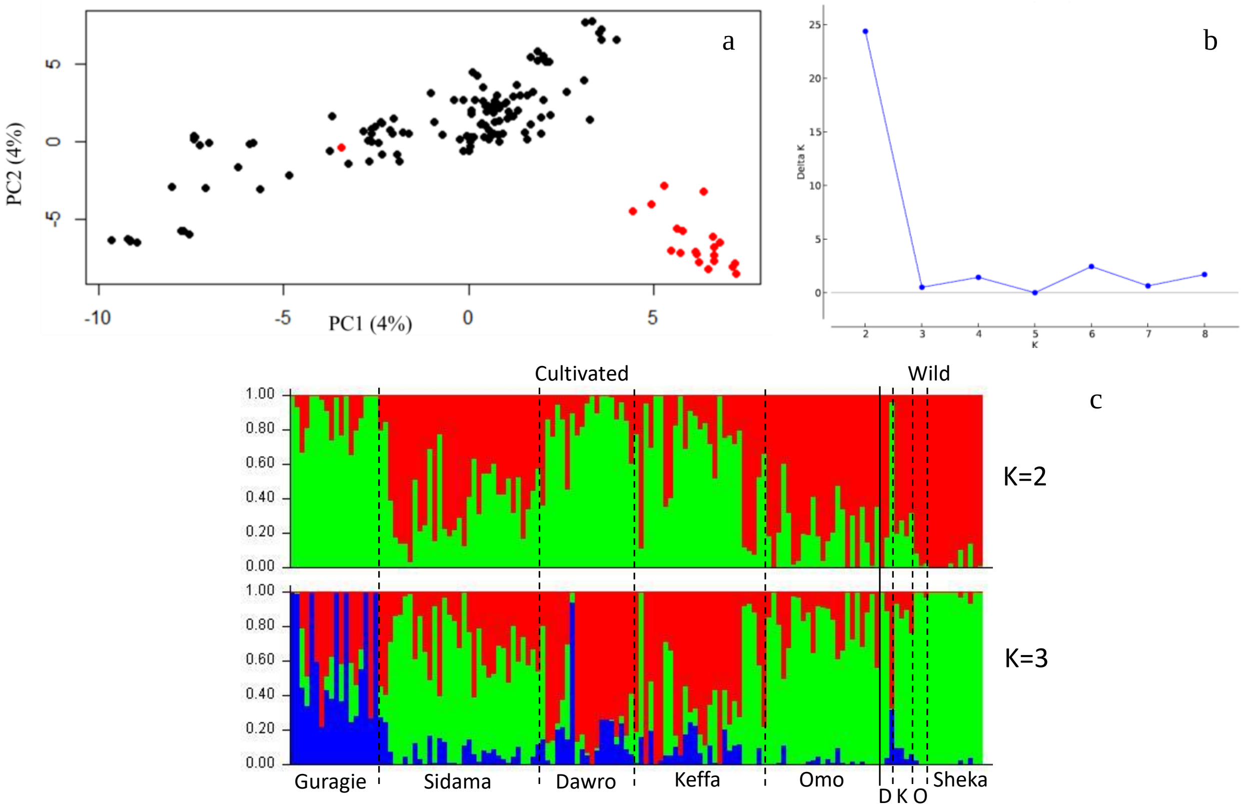

Supplementary Fig.9. Analysis of Ethiopian cultivated and wild enset population diversity and structure using 5011 neutral SNP markers. a) PCA analysis of 120 cultivated (black) and 21 wild (red) enset accessions. b) Evanno plot of Delta K calculated from K ranging from 2 to 9 analyzed using Structure-Harvester. c) Population structure analysis of cultivated (left) and wild (right) enset. Accessions are grouped first by cultivated or wild (separated by continuous vertical line) and then by their regions of origin (separated by dashed line). D = Dawro; K = Keffa; O = Omo.
