# Supplementary material for: Accumulation of mutations in genes associated with sexual reproduction contributed to the domestication of a vegetatively propagated staple crop, enset
Source: Hortic Res. 2020 Nov 1;7:185. doi: 10.1038/s41438-020-00409-7 (PMC7603512; doi:10.1038/s41438-020-00409-7)
Supplement: Supplementary file 10 — Supplementary Fig.10 [file 41438_2020_409_MOESM10_ESM.pdf]

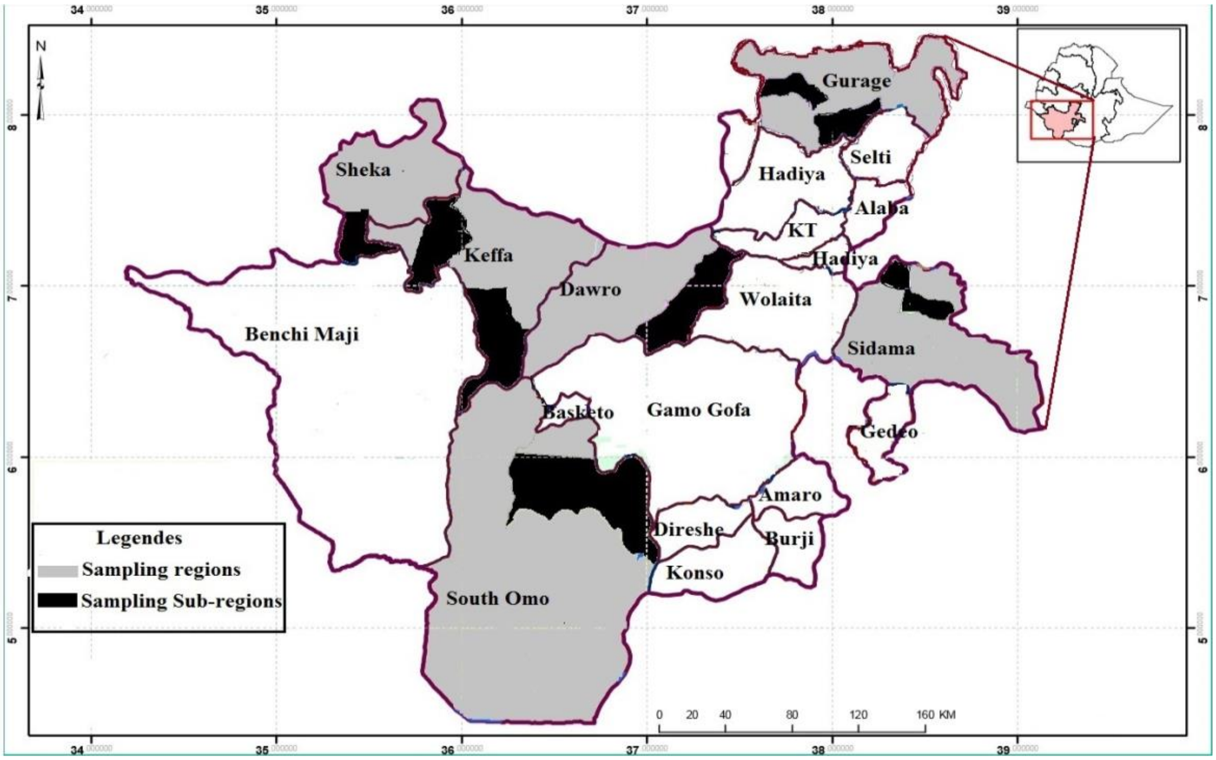

Supplementary Fig.10 Sampling regions (highlighted in grey and purple perimeter) and sub-regions (highlighted in black) in Southern Nation, Nationalities and People Region (SNNPR), Ethiopia.
