# Supplementary material for: Accumulation of mutations in genes associated with sexual reproduction contributed to the domestication of a vegetatively propagated staple crop, enset
Source: Hortic Res. 2020 Nov 1;7:185. doi: 10.1038/s41438-020-00409-7 (PMC7603512; doi:10.1038/s41438-020-00409-7)
Supplement: Supplementary file 11 — Supplementary Table 1 [file 41438_2020_409_MOESM11_ESM.pdf]

Supplementary Table 1: Total number of filtered and unfiltered SNP markers distributed across 11 chromosomes and chr_unknown (SNPs within contigs that had not been assigned to a chromosome in the assembly).

**Chromosome**	**Number of SNPs**	
	**Unfiltered**	**Filtered**
1	1644	422
2	1262	251
3	1801	387
4	1978	462
5	1702	399
6	2056	417
7	1732	410
8	1955	442
9	1709	396
10	1708	368
11	1536	327
ChrUn_random	3800	887
Total	22883	5169
