# Supplementary material for: Accumulation of mutations in genes associated with sexual reproduction contributed to the domestication of a vegetatively propagated staple crop, enset
Source: Hortic Res. 2020 Nov 1;7:185. doi: 10.1038/s41438-020-00409-7 (PMC7603512; doi:10.1038/s41438-020-00409-7)
Supplement: Supplementary file 12 — Supplementary Table 2 [file 41438_2020_409_MOESM12_ESM.pdf]

Supplementary Table 2: Number and proportion of nucleotide substitution types within the identified SNPs

**Allele**	**Number**	**Proportion (%)**
A/C	567	10.97
A/G	1502	29.06
A/T	431	8.34
C/G	504	9.75
C/T	1449	28.03
G/T	580	11.22
multiple allele	136	2.63
Total	5169	100
