# Supplementary material for: Accumulation of mutations in genes associated with sexual reproduction contributed to the domestication of a vegetatively propagated staple crop, enset
Source: Hortic Res. 2020 Nov 1;7:185. doi: 10.1038/s41438-020-00409-7 (PMC7603512; doi:10.1038/s41438-020-00409-7)
Supplement: Supplementary file 13 — Supplementary Table 3 [file 41438_2020_409_MOESM13_ESM.pdf]

Supplementary Table 3: List of genes that contain outlier SNP markers which differenciate wild and cultivated enset identified using Lositan. Genes hihghligted in grey were deemed significant by Lositan and pcadapt.

NO	Gene-ID	Chro	Start	End	Gene/Protein name
1	GSMUA_Achr1G14390_	1	1E+07	1E+07	EARLY FLOWERING 3
2	GSMUA_Achr1G17150_	1	1E+07	1E+07	Transcription factor GAMYB
3	GSMUA_Achr1G24430_	1	2E+07	2E+07	WD domain G-beta repeat domain containing protein
4	GSMUA_Achr2G05290_	2	1E+07	1E+07	uncharacterized protein
5	GSMUA_Achr2G14580_	2	2E+07	2E+07	Guanine nucleotide-binding protein subunit beta-like protein
6	GSMUA_Achr2G15650_	2	2E+07	2E+07	CK1_CaseinKinase_1a.5
7	GSMUA_Achr2G15820_	2	2E+07	2E+07	LRR receptor-like serine/threonine-protein kinase
8	GSMUA_Achr2G15900_	2	2E+07	2E+07	Hypothetical protein
9	GSMUA_Achr2G22590_	2	2E+07	2E+07	Lipid phosphate phosphatase
10	GSMUA_Achr2G22670_	2	2E+07	2E+07	expressed protein
11	GSMUA_Achr3G09010_	3	7E+06	7E+06	Serine carboxypeptidase II-3
12	GSMUA_Achr3G14810_	3	2E+07	2E+07	serine/threonine-protein kinase NAK
13	GSMUA_Achr3G16360_	3	2E+07	2E+07	E3 ubiquitin-protein ligase SINAT5
14	GSMUA_Achr3G17890_	3	2E+07	2E+07	Alpha-taxilin
15	GSMUA_Achr3G19020_	3	2E+07	2E+07	DUF630/DUF632 domains containing
16	GSMUA_Achr3G19350_	3	2E+07	2E+07	DNA repair protein recA homolog
17	GSMUA_Achr3G19710_	3	2E+07	2E+07	Galactosylgalactosylxylosylprotein 3-beta-glucuronosyltransferase 1
18	GSMUA_Achr3G21200_	3	2E+07	2E+07	Seryl-tRNA synthetase
19	GSMUA_Achr3G22370_	3	2E+07	2E+07	DDB1- and CUL4-associated factor homolog 1
20	GSMUA_Achr3G22960_	3	2E+07	2E+07	expressed protein
21	GSMUA_Achr3G23210_	3	2E+07	2E+07	expressed protein
22	GSMUA_Achr3G31400_	3	3E+07	3E+07	expressed protein
23	GSMUA_Achr4G05390_	4	4E+06	4E+06	elongation factor
24	GSMUA_Achr4G05620_	4	4E+06	4E+06	uncharacterized protein
25	GSMUA_Achr4G06740_	4	5E+06	5E+06	AP-3 complex
26	GSMUA_Achr4G09960_	4	7E+06	7E+06	aarF domain-containing protein kinase
27	GSMUA_Achr4G13320_	4	1E+07	1E+07	Pentatricopeptide repeat-containing protein
28	GSMUA_Achr4G16420_	4	1E+07	1E+07	expressed protein
29	GSMUA_Achr4G17010_	4	2E+07	2E+07	ABC tranporter B family mmber 2
30	GSMUA_Achr4G19410_	4	2E+07	2E+07	Dicer-like protein 2
31	GSMUA_Achr4G26350_	4	3E+07	3E+07	Calcineurin B-like protein 1
32	GSMUA_Achr5G08230_	5	6E+06	6E+06	Two-component response regulator ARR9
33	GSMUA_Achr5G08470_	5	6E+06	6E+06	Phosphoinositide phospholipase C6
34	GSMUA_Achr5G10830_	5	8E+06	8E+06	expressed protein
35	GSMUA_Achr5G25780_	5	3E+07	3E+07	Cytochrome P450
36	GSMUA_Achr5G29540_	5	3E+07	3E+07	uncharacterized protein

37	GSMUA_Achr5G29680_	5	3E+07	3E+07	inactive leucine-rich repeat receptor-like protein kinase
38	GSMUA_Achr6G00910_	6	6E+05	6E+05	Ent-kaurene oxidase
39	GSMUA_Achr6G03360_	6	2E+06	2E+06	Sulfate transporter 1.3
40	GSMUA_Achr6G04770_	6	3E+06	3E+06	uncharacterized protein
41	GSMUA_Achr6G11730_	6	8E+06	8E+06	Nudix hydrolase
42	GSMUA_Achr6G11920_	6	8E+06	8E+06	Fanconi-associated nuclease 1 homolog
43	GSMUA_Achr6G12410_	6	8E+06	8E+06	Beta-glucosidase 11
44	GSMUA_Achr6G13180_	6	9E+06	9E+06	Hypothetical protein
45	GSMUA_Achr6G13960_	6	9E+06	9E+06	DNA replication licensing factor mcm6
46	GSMUA_Achr6G23760_	6	2E+07	2E+07	uncharacterized protein
47	GSMUA_Achr6G31260_	6	3E+07	3E+07	single myb
48	GSMUA_Achr7G03050_	7	2E+06	2E+06	glycosyl transferase
49	GSMUA_Achr7G03500_	7	3E+06	3E+06	expressed protein
50	GSMUA_Achr7G06340_	7	5E+06	5E+06	Glucan endo-C3-beta-glucosidase 8
51	GSMUA_Achr7G09240_	7	8E+06	8E+06	ALWAYS EARLY 3
52	GSMUA_Achr7G14910_	7	1E+07	1E+07	kDa class I heat shock protein
53	GSMUA_Achr7G15510_	7	1E+07	1E+07	elongation factor 1-alpha
54	GSMUA_Achr7G24290_	7	3E+07	3E+07	Ubiquitin carboxyl-terminal hydrolase 6
55	GSMUA_Achr7G25050_	7	3E+07	3E+07	LRR receptor-like serine/threonine-protein kinase
56	GSMUA_Achr7G25860_	7	3E+07	3E+07	Zinc finger CCCH domain-containing protein 54
57	GSMUA_Achr7G26900_	7	3E+07	3E+07	Hypothetical protein
58	GSMUA_Achr8G00590_	8	5E+05	5E+05	Hypothetical protein
59	GSMUA_Achr8G13290_	8	1E+07	1E+07	Whole genome shotgun sequence of line
60	GSMUA_Achr8G22320_	8	3E+07	3E+07	ATP-dependent Clp protease proteolytic subunit
61	GSMUA_Achr8G25610_	8	3E+07	3E+07	inactive leucine-rich repeat receptor-like protein kinase
62	GSMUA_Achr8G26780_	8	3E+07	3E+07	Type I inositol-trisphosphate 5-phosphatase CVP2
63	GSMUA_Achr8G27350_	8	3E+07	3E+07	DEAD-box ATP-dependent RNA helicase 35A
64	GSMUA_Achr8G28760_	8	3E+07	3E+07	Cytosolic Fe-S cluster assembly factor nubp1
65	GSMUA_Achr8G30140_	8	3E+07	3E+07	methyltransferase PMT26
66	GSMUA_Achr9G04390_	9	3E+06	3E+06	Whole genome shotgun sequence of line
67	GSMUA_Achr9G07510_	9	5E+06	5E+06	F-box protein
68	GSMUA_Achr9G07550_	9	5E+06	5E+06	lysine ketoglutarate ketog lutarate reductase trans-splicing related
69	GSMUA_Achr9G16720_	9	1E+07	1E+07	calcium-activated outward-rectifying related
70	GSMUA_Achr9G17450_	9	1E+07	1E+07	Sugar transporter ERD6-like 5
71	GSMUA_Achr9G18630_	9	1E+07	1E+07	expressed protein

72	GSMUA_Achr9G22250_	9	3E+07	3E+07	Predicted protein
73	GSMUA_Achr9G24060_	9	3E+07	3E+07	GDSL esterase/lipase
74	GSMUA_Achr10G0033(	10	9E+05	9E+05	exonuclease putative
75	GSMUA_Achr10G0479(	10	1E+07	1E+07	Alpha-soluble NSF attachment expressed protein
76	GSMUA_Achr10G0506(	10	1E+07	1E+07	expressed protein
77	GSMUA_Achr10G1314(	10	2E+07	2E+07	Serine/threonine-protein kinase PBS1
78	GSMUA_Achr10G1674(	10	2E+07	2E+07	dual specificity protein phosphatase
79	GSMUA_Achr10G1693(	10	2E+07	2E+07	uncharacterized protein
80	GSMUA_Achr10G1893(	10	3E+07	3E+07	Developmentally-regulated GTP-binding protein 1
81	GSMUA_Achr10G2009(	10	3E+07	3E+07	glycosyltransferase
82	GSMUA_Achr10G2211(	10	3E+07	3E+07	Hypothetical protein
83	GSMUA_Achr10G2564(	10	3E+07	3E+07	Patatin group A-3
84	GSMUA_Achr10G2590(	10	3E+07	3E+07	Homeobox-leucine zipper protein ROC8
85	GSMUA_Achr10G2599(	10	3E+07	3E+07	Ubiquitin-conjugating enzyme E2 variant 1A
86	GSMUA_Achr10G2869(	10	3E+07	3E+07	stress-induced protein
87	GSMUA_Achr10G2881(	10	3E+07	3E+07	potasium efflux antiporter
88	GSMUA_Achr11G0677(	11	5E+06	5E+06	DNA-binding protein SMUBP-2
89	GSMUA_Achr11G1375(	11	1E+07	1E+07	NPL4-like protein
