# Supplementary material for: Accumulation of mutations in genes associated with sexual reproduction contributed to the domestication of a vegetatively propagated staple crop, enset
Source: Hortic Res. 2020 Nov 1;7:185. doi: 10.1038/s41438-020-00409-7 (PMC7603512; doi:10.1038/s41438-020-00409-7)
Supplement: Supplementary file 14 — Supplementary Table 4 [file 41438_2020_409_MOESM14_ESM.pdf]

Supplementary Table 4: Genes under selection during enset domestication associated to flowering, seed development and germination. Genes hihghligted in grey were deemed significant by Lositan and pcadapt.

Gene_ID	Chrom	Position		Gene/Protein name	Function	Reference
		Start	End			
GSMUA_Achr1G14390_001	1	10946319	10951750	EARLY FLOWERING 3	Regulates the frequency and extent of photoperiodism and flowering	https://www.uniprot.org/uniprot/M0RZU7
GSMUA_Achr9G04390_001	9	2928750	2930449	Whole genome shotgun sequence of line	Embryo development ending in seed dormancy	https://www.uniprot.org/uniprot/M0TXE8
GSMUA_Achr4G16420_001	4	14853262	14858031	Expressed protein	Negative regulation of photomorphogenesis. Three stages of plant development where photomorphogenesis occurs: seed germination, seedling development, and the switch from the vegetative to the flowering stage	https://www.uniprot.org/uniprot/M0SPF6
GSMUA_Achr8G28760_001	8	31455952	31458272	Cytosolic Fe-S cluster assembly factor nubp1	Essential for embryo development	https://www.uniprot.org/uniprot/M0TUH9
GSMUA_Achr3G21200_001	3	22211785	22217055	AA_TRNA_LIGASE_II domain-containing protein	Innvolve in plant ovule development	https://www.uniprot.org/uniprot/M0SGI6
GSMUA_Achr7G24290_001	7	26261072	26277219	Ubiquitin carboxyl-terminal hydrolase 6	Regulate seed size by promoting cell proliferation in the integuments of ovules and developing seeds in Arabdopsis	https://www.uniprot.org/uniprot/Q9FPS9
GSMUA_Achr2G15820_001	2	16961306	16967890	LRR receptor-like serine/threonine-protein kinase	Regulates plants inflorescence architecture and conferes bacterial resistance	https://www.uniprot.org/uniprot/Q42371
GSMUA_Achr2G14580_001	2	16286743	16288080	WD_REPEATS_REGION domain-containing protein	Regulation of flowering and embyro development ending in seed dormancy	https://www.uniprot.org/uniprot/I1HV81
GSMUA_Achr3G22370_001	3	23336294	23345340	DDB1- and CUL4-associated factor homolog 1	Required for plant embryogenesis and ffect several other developmental processes including leaf, shoot, and flower development	https://www.uniprot.org/uniprot/Q9M086
GSMUA_Achr3G14810_001	3	15195572	15197603	Serine/threonine-protein kinase NAK	Regulates flowering time by modulating the photoperiod pathway	https://www.uniprot.org/uniprot/Q944Q0
GSMUA_Achr1G17150_001	1	12797871	12807633	Transcription factor GAMYB/Myb-like protein domain	Involved in pollen and floral organs development	https://www.uniprot.org/uniprot/Q0JIC2&format=html
GSMUA_Achr6G00910_001	6	620666	628658	Ent-kaurene oxidase	Catalises Gibberellin (GA) Biosynthesis process which which are important for seed germination, stem elongation, flowering, and fruit set	Swain et al.,2005
GSMUA_Achr5G08230_001	5	5965616	5967602	Two-component response regulator ARR9	Play a role in modulating circadian rhythms	Ishida K, et al (2008)
GSMUA_Achr2G15650_001	2	16851733	16856529	CK1_CaseinKinase_1	Play key role in regulating plant flowering	Kang et al.,2020; Uehara et a.,2019
GSMUA_Achr9G24060_001	9	28897340	28899954	GDSL esterase/lipase	Affects plant leaf expansion, hypocotyl elongation and seed growth	Ma et al.,2018
GSMUA_Achr10G25990_001	10	29754371	29757603	Ubiquitin-conjugating enzyme E2 variant 1A	Important for seed size and germination	Wen et al.,2008; Li and Li 2014
GSMUA_Achr8G26780_001	8	30196141	30199104	Type I inositol-trisphosphate 5-phosphatase CVP2	Regulate and maintain seedling growth and pollen dormancy	Zhong, Ruiqin et al.,2004; Gunesekera, Bhadra et al.,2007
GSMUA_Achr5G25780_001	5	26746251	26747873	Cytochrome P450	Regulates a domestication trait in cultivated tomato	Chakrabarti et al,2013
GSMUA_Achr7G25860_001	7	27368961	27371264	Zinc finger CCCH domain-containing protein 54	Confers delayed senescence and stress tolerance and plant drowth, and selected during domestication	Jan et al.,2013; Li et al.,2017
GSMUA_Achr3G16360_001	3	17578436	17582250	E3 ubiquitin-protein ligase SINAT5	Positively regulating flowering time	Park et al. (2010a)
