# Supplementary material for: Accumulation of mutations in genes associated with sexual reproduction contributed to the domestication of a vegetatively propagated staple crop, enset
Source: Hortic Res. 2020 Nov 1;7:185. doi: 10.1038/s41438-020-00409-7 (PMC7603512; doi:10.1038/s41438-020-00409-7)
Supplement: Supplementary file 15 — Supplementary Table 5 [file 41438_2020_409_MOESM15_ESM.pdf]

Supplementary Table 5: Cluster assignment of enset accessions by DAPC analysis using all 5169 GBS-based SNP markers and 5011 neutral SNP markers. Acessions highlighted in grey are those found two switch cluster when using different SNP datasets

ID	Category	Region	Area of collection	All SNPs	Neutral
01g	Domestic	Dawro	Cultivated and wild	1	1
02g	Domestic	Guragie	Cultivated only	1	1
03d	Domestic	Keffa	Cultivaed and wild	1	1
03g	Domestic	Guragie	Cultivated only	1	1
06d	Domestic	Dawro	Cultivated and wild	1	1
07g	Domestic	Guragie	Cultivated only	1	1
08g	Domestic	Guragie	Cultivated only	1	1
09d	Domestic	Dawro	Cultivated and wild	1	1
09g	Domestic	Guragie	Cultivated only	1	1
14g	Domestic	Guragie	Cultivated only	1	1
15g	Domestic	Guragie	Cultivated only	1	1
16g	Domestic	Guragie	Cultivated only	1	1
18g	Domestic	Guragie	Cultivated only	1	1
19g	Domestic	Guragie	Cultivated only	1	1
20g	Domestic	Guragie	Cultivated only	1	1
22g	Domestic	Guragie	Cultivated only	1	1
23g	Domestic	Guragie	Cultivated only	1	1
25g	Domestic	Guragie	Cultivated only	1	1
26g	Domestic	Guragie	Cultivated only	1	1
27d	Domestic	Dawro	Cultivated and wild	1	1
28d	Domestic	Dawro	Cultivated and wild	1	1
29d	Domestic	Dawro	Cultivated and wild	1	1
29g	Domestic	Guragie	Cultivated only	1	1
30d	Wild	Dawro	Cultivated and wild	1	1
35d	Domestic	Dawro	Cultivated and wild	1	1
05k	Domestic	Guragie	Cultivated only	2	1
15h	Domestic	Sidama	Cultivated only	2	1
21g	Domestic	Guragie	Cultivated only	2	1
23h	Domestic	Sidama	Cultivated only	2	1
26h	Domestic	Sidama	Cultivated only	2	1
28g	Domestic	Guragie	Cultivated only	2	1
01d	Domestic	Dawro	Cultivaed and wild	2	2
01h	Domestic	Sidama	Cultivated only	2	2
02d	Domestic	Dawro	Cultivaed and wild	2	2
02h	Domestic	Sidama	Cultivated only	2	2
03h	Domestic	Sidama	Cultivated only	2	2
03ho	Domestic	Keffa	Cultivaed and wild	2	2
03k	Domestic	Keffa	Cultivaed and wild	2	2
03m	Domestic	Omo	Cultivaed and wild	2	2
04h	Domestic	Sidama	Cultivated only	2	2
05m	Domestic	Omo	Cultivaed and wild	2	2
06h	Domestic	Sidama	Cultivated only	2	2
06m	Domestic	Omo	Cultivaed and wild	2	2
07h	Domestic	Sidama	Cultivated only	2	2
08d	Domestic	Keffa	Cultivaed and wild	2	2
08h	Domestic	Sidama	Cultivated only	2	2
08m	Domestic	Omo	Cultivaed and wild	2	2

09h	Domestic	Sidama	Cultivated only	2	2
09ho	Domestic	Keffa	Cultivaed and wild	2	2
10d	Domestic	Keffa	Cultivaed and wild	2	2
10m	Domestic	Omo	Cultivaed and wild	2	2
11m	Domestic	Omo	Cultivaed and wild	2	2
12g	Domestic	Keffa	Cultivaed and wild	2	2
12h	Domestic	Sidama	Cultivated only	2	2
12k	Domestic	Keffa	Cultivaed and wild	2	2
12m	Domestic	Omo	Cultivaed and wild	2	2
13h	Domestic	Sidama	Cultivated only	2	2
14h	Domestic	Sidama	Cultivated only	2	2
15d	Domestic	Keffa	Cultivaed and wild	2	2
15m	Domestic	Omo	Cultivaed and wild	2	2
16d	Domestic	Keffa	Cultivaed and wild	2	2
16h	Domestic	Sidama	Cultivated only	2	2
18h	Domestic	Sidama	Cultivated only	2	2
19k	Domestic	Keffa	Cultivaed and wild	2	2
19m	Domestic	Omo	Cultivaed and wild	2	2
20h	Domestic	Sidama	Cultivated only	2	2
20k	Domestic	Keffa	Cultivaed and wild	2	2
21d	Domestic	Keffa	Cultivaed and wild	2	2
21k	Domestic	Keffa	Cultivaed and wild	2	2
25d	Domestic	Keffa	Cultivaed and wild	2	2
25k	Domestic	Keffa	Cultivaed and wild	2	2
27k	Domestic	Keffa	Cultivaed and wild	2	2
28k	Domestic	Keffa	Cultivaed and wild	2	2
30m	Domestic	Omo	Cultivaed and wild	2	2
31h	Domestic	Sidama	Cultivated only	2	2
31k	Domestic	Keffa	Cultivaed and wild	2	2
31m	Domestic	Omo	Cultivaed and wild	2	2
32h	Domestic	Sidama	Cultivated only	2	2
32k	Domestic	Keffa	Cultivaed and wild	2	2
33d	Domestic	Keffa	Cultivaed and wild	2	2
33h	Domestic	Sidama	Cultivated only	2	2
33k	Domestic	Keffa	Cultivaed and wild	2	2
34k	Domestic	Keffa	Cultivaed and wild	2	2
36h	Domestic	Sidama	Cultivated only	2	2
36k	Domestic	Keffa	Cultivaed and wild	2	2
36m	Domestic	Omo	Cultivaed and wild	2	2
37h	Domestic	Sidama	Cultivated only	2	2
38h	Domestic	Sidama	Cultivated only	2	2
38k	Domestic	Keffa	Cultivaed and wild	2	2
38m	Domestic	Omo	Cultivaed and wild	2	2
39h	Domestic	Sidama	Cultivated only	2	2
39k	Domestic	Keffa	Cultivaed and wild	2	2
40m	Domestic	Dawro	Cultivated and wild	2	2
41h	Domestic	Sidama	Cultivated only	2	2
41m	Domestic	Dawro	Cultivated and wild	2	2
42h	Domestic	Sidama	Cultivated only	2	2
42m	Domestic	Dawro	Cultivated and wild	2	2
43h	Domestic	Sidama	Cultivated only	2	2
44h	Domestic	Sidama	Cultivated only	2	2

44k	Domestic	Keffa	Cultivaed and wild	2	2
44m	Domestic	Dawro	Cultivated and wild	2	2
45k	Domestic	Omo	Cultivaed and wild	2	2
45m	Domestic	Dawro	Cultivated and wild	2	2
46h	Domestic	Sidama	Cultivated only	2	2
46m	Domestic	Dawro	Cultivated and wild	2	2
47k	Domestic	Omo	Cultivaed and wild	2	2
47m	Domestic	Dawro	Cultivated and wild	2	2
48h	Domestic	Sidama	Cultivated only	2	2
48k	Domestic	Omo	Cultivaed and wild	2	2
49h	Domestic	Sidama	Cultivated only	2	2
49m	Domestic	Dawro	Cultivated and wild	2	2
50h	Domestic	Sidama	Cultivated only	2	2
50m	Domestic	Dawro	Cultivated and wild	2	2
51K	Domestic	Omo	Cultivaed and wild	2	2
52m	Domestic	Guragie	Cultivated only	2	2
53K	Domestic	Omo	Cultivaed and wild	2	2
54K	Domestic	Omo	Cultivaed and wild	2	2
55k	Domestic	Omo	Cultivaed and wild	2	2
56k	Domestic	Omo	Cultivaed and wild	2	2
61k	Domestic	Omo	Cultivaed and wild	2	2
63k	Domestic	Omo	Cultivaed and wild	2	2
01b	Wild	Sheka	Wild	3	3
01s	Wild	Sheka	Wild	3	3
02b	Wild	Sheka	Wild	3	3
03b	Wild	Sheka	Wild	3	3
03s	Wild	Sheka	Wild	3	3
04b	Wild	Sheka	Wild	3	3
04d	Wild	Dawro	Wild	3	3
04s	Wild	Sheka	Wild	3	3
07K	Wild	Keffa	Wild	3	3
07s	Wild	Sheka	Wild	3	3
09s	Wild	Sheka	Wild	3	3
17d	Wild	Dawro	Wild	3	3
17m	Wild	Omo	Wild	3	3
23m	Wild	Omo	Wild	3	3
24k	Wild	Keffa	Wild	3	3
39m	Wild	Omo	Wild	3	3
42k	Wild	Keffa	Wild	3	3
43k	Wild	Keffa	Wild	3	3
60k	Wild	Keffa	Wild	3	3
6s	Wild	Sheka	Wild	3	3
