# Supplementary material for: Accumulation of mutations in genes associated with sexual reproduction contributed to the domestication of a vegetatively propagated staple crop, enset
Source: Hortic Res. 2020 Nov 1;7:185. doi: 10.1038/s41438-020-00409-7 (PMC7603512; doi:10.1038/s41438-020-00409-7)
Supplement: Supplementary file 16 — Supplementary Table 6 [file 41438_2020_409_MOESM16_ESM.pdf]

Supplementary Table 6: Cluster assignment of enset accessions by STRUCTURE analysis using all 5169 GBS-based SNP markers and 5011 neutral SNP markers.

Acessions highlighted in grey are those found two switch cluster when using different SNP

Accession information				K=2		K=3	
**ID**	**Category**	**Region**	**Area of collection**	**All SNPs**	**Neutral**	**All SNPs**	**Neutral**
01b	Wild	Sheka	Wild	2	2	1	1
01d	Cultivated	Dawro	Cultivated and wild	1	1	2	2
01g	Cultivated	Dawro	Cultivated and wild	1	1	3	3
01h	Cultivated	Sidama	Cultivated only	2	2	1	1
01s	Wild	Sheka	Wild	2	2	1	1
02b	Wild	Sheka	Wild	2	2	1	1
02d	Cultivated	Dawro	Cultivated and wild	1	1	2	2
02g	Cultivated	Guragie	Cultivated only	1	1	3	3
02h	Cultivated	Sidama	Cultivated only	2	2	1	1
03b	Wild	Sheka	Wild	2	2	1	1
03d	Cultivated	Keffa	Cultivated and wild	1	1	2	2
03g	Cultivated	Guragie	Cultivated only	1	1	Uncluster	Unclustered
03h	Cultivated	Sidama	Cultivated only	2	2	1	1
03ho	Cultivated	Keffa	Cultivated and wild	1	1	2	2
03k	Cultivated	Keffa	Cultivated and wild	1	1	2	2
03m	Cultivated	Omo	Cultivated and wild	2	2	1	1
03s	Wild	Sheka	Wild	2	2	1	1
04b	Wild	Sheka	Wild	2	2	1	1
04d	Wild	Dawro	Cultivated and wild	2	2	1	1
04h	Cultivated	Sidama	Cultivated only	2	2	1	1
04s	Wild	Sheka	Wild	2	2	1	1
05k	Cultivated	Guragie	Cultivated only	2	2	1	1
05m	Cultivated	Omo	Cultivated and wild	2	2	1	1
06d	Cultivated	Dawro	Cultivated and wild	1	1	2	2
06h	Cultivated	Sidama	Cultivated only	2	2	1	1
06m	Cultivated	Omo	Cultivated and wild	2	2	1	1
07g	Cultivated	Guragie	Cultivated only	1	1	2	Unclustered
07h	Cultivated	Sidama	Cultivated only	1	1	Uncluster	Unclustered
07K	Wild	Keffa	Wild	2	2	1	1
07s	Wild	Sheka	Wild	2	2	1	1
08d	Cultivated	Keffa	Cultivated and wild	1	2	Uncluster	1
08g	Cultivated	Guragie	Cultivated only	1	1	3	3
08h	Cultivated	Sidama	Cultivated only	2	2	1	1
08m	Cultivated	Omo	Cultivated and wild	1	1	2	2
09d	Cultivated	Dawro	Cultivated and wild	1	1	3	3
09g	Cultivated	Guragie	Cultivated only	1	1	3	3
09h	Cultivated	Sidama	Cultivated only	2	2	1	1
09ho	Cultivated	Keffa	Cultivated and wild	1	1	2	2
09s	Wild	Sheka	Wild	2	2	1	1
10d	Cultivated	Keffa	Cultivated and wild	1	1	2	2
10m	Cultivated	Omo	Cultivated and wild	2	2	1	1
11m	Cultivated	Omo	Cultivated and wild	2	2	1	1
12g	Cultivated	Keffa	Cultivated and wild	1	1	2	2
12h	Cultivated	Sidama	Cultivated only	1	1	2	2
12k	Cultivated	Keffa	Cultivated and wild	1	1	2	2
12m	Cultivated	Omo	Cultivated and wild	2	2	1	1
13h	Cultivated	Sidama	Cultivated only	2	2	1	1

14g	Cultivated	Guragie	Cultivated only	1	1	2	Unclustered
14h	Cultivated	Sidama	Cultivated only	1	1	2	2
15d	Cultivated	Keffa	Cultivated and wild	1	1	2	2
15g	Cultivated	Guragie	Cultivated only	1	1	Uncluster	Unclustered
15h	Cultivated	Sidama	Cultivated only	2	2	1	1
15m	Cultivated	Omo	Cultivated and wild	2	2	1	1
16d	Cultivated	Keffa	Cultivated and wild	1	1	2	2
16g	Cultivated	Guragie	Cultivated only	1	1	3	3
16h	Cultivated	Sidama	Cultivated only	2	2	1	1
17d	Wild	Dawro	Cultivated and wild	2	2	1	1
17m	Wild	Omo	Wild	2	2	1	1
18g	Cultivated	Guragie	Cultivated only	1	1	Uncluster	Unclustered
18h	Cultivated	Sidama	Cultivated only	2	2	1	1
19g	Cultivated	Guragie	Cultivated only	1	1	3	3
19k	Cultivated	Keffa	Cultivated and wild	1	1	2	2
19m	Cultivated	Omo	Cultivated and wild	2	2	1	1
20g	Cultivated	Guragie	Cultivated only	1	1	Uncluster	Unclustered
20h	Cultivated	Sidama	Cultivated only	2	2	1	1
20k	Cultivated	Keffa	Cultivated and wild	1	1	2	2
21d	Cultivated	Keffa	Cultivated and wild	1	1	2	2
21g	Cultivated	Guragie	Cultivated only	1	1	2	2
21k	Cultivated	Keffa	Cultivated and wild	1	1	2	2
22g	Cultivated	Guragie	Cultivated only	1	1	3	3
23g	Cultivated	Guragie	Cultivated only	1	1	3	3
23h	Cultivated	Sidama	Cultivated only	2	2	1	1
23m	Wild	Omo	Wild	2	2	1	1
24k	Wild	Keffa	Wild	2	2	1	1
25d	Cultivated	Keffa	Cultivated and wild	1	1	2	2
25g	Cultivated	Guragie	Cultivated only	1	1	2	2
25k	Cultivated	Keffa	Cultivated and wild	2	2	1	1
26g	Cultivated	Guragie	Cultivated only	1	1	3	3
26h	Cultivated	Sidama	Cultivated only	2	2	1	1
27d	Cultivated	Dawro	Cultivated and wild	1	1	2	2
27k	Cultivated	Keffa	Cultivated and wild	2	2	1	1
28d	Cultivated	Dawro	Cultivated and wild	1	1	2	2
28g	Cultivated	Guragie	Cultivated only	1	1	2	2
28k	Cultivated	Keffa	Cultivated and wild	1	1	2	2
29d	Cultivated	Dawro	Cultivated and wild	1	1	2	2
29g	Cultivated	Guragie	Cultivated only	1	1	2	2
30d	Cultivated	Dawro	Cultivated and wild	1	1	2	2
30m	Cultivated	Omo	Cultivated and wild	2	2	1	1
31h	Cultivated	Sidama	Cultivated only	1	1	2	2
31k	Cultivated	Keffa	Cultivated and wild	1	1	2	2
31m	Cultivated	Omo	Cultivated and wild	2	2	1	1
32h	Cultivated	Sidama	Cultivated only	2	2	1	1
32k	Cultivated	Keffa	Cultivated and wild	1	1	2	2
33d	Cultivated	Keffa	Cultivated and wild	1	1	2	2
33h	Cultivated	Sidama	Cultivated only	1	1	2	1
33k	Cultivated	Keffa	Cultivated and wild	1	1	2	2
34k	Cultivated	Keffa	Cultivated and wild	1	1	2	2
35d	Wild	Dawro	Cultivated and wild	1	1	2	2
36h	Cultivated	Sidama	Cultivated only	1	1	2	2

36k	Cultivated	Keffa	Cultivated and wild	1	1	2	2
36m	Cultivated	Omo	Cultivated and wild	2	2	1	1
37h	Cultivated	Sidama	Cultivated only	1	1	2	Unclustered
38h	Cultivated	Sidama	Cultivated only	1	2	1	1
38k	Cultivated	Keffa	Cultivated and wild	1	1	2	2
38m	Cultivated	Omo	Cultivated and wild	2	2	1	1
39h	Cultivated	Sidama	Cultivated only	1	2	1	1
39k	Cultivated	Keffa	Cultivated and wild	1	1	2	2
39m	Wild	Omo	Wild	2	2	1	1
40m	Cultivated	Dawro	Cultivated and wild	2	2	1	1
41h	Cultivated	Sidama	Cultivated only	1	1	2	2
41m	Cultivated	Dawro	Cultivated and wild	1	2	Uncluster	1
42h	Cultivated	Sidama	Cultivated only	2	2	1	1
42k	Wild	Keffa	Wild	2	2	1	1
42m	Cultivated	Dawro	Cultivated and wild	2	2	1	1
43h	Cultivated	Sidama	Cultivated only	1	1	Uncluster	Unclustered
43k	Wild	Keffa	Wild	2	2	1	1
44h	Cultivated	Sidama	Cultivated only	2	2	1	1
44k	Cultivated	Keffa	Cultivated and wild	1	1	2	2
44m	Cultivated	Dawro	Cultivated and wild	2	2	1	1
45k	Cultivated	Omo	Cultivated and wild	1	1	2	2
45m	Cultivated	Dawro	Cultivated and wild	2	2	1	1
46h	Cultivated	Sidama	Cultivated only	2	2	1	1
46m	Cultivated	Dawro	Cultivated and wild	2	2	1	1
47k	Cultivated	Omo	Cultivated and wild	1	1	2	2
47m	Cultivated	Dawro	Cultivated and wild	2	2	1	1
48h	Cultivated	Sidama	Cultivated only	1	2	1	1
48k	Cultivated	Omo	Cultivated and wild	1	1	2	2
49h	Cultivated	Sidama	Cultivated only	1	1	2	Unclustered
49m	Cultivated	Dawro	Cultivated and wild	2	2	1	1
50h	Cultivated	Sidama	Cultivated only	2	2	1	1
50m	Cultivated	Dawro	Cultivated and wild	2	2	1	1
51K	Cultivated	Omo	Cultivated and wild	1	1	2	2
52m	Cultivated	Guragie	Cultivated only	2	2	1	1
53K	Cultivated	Omo	Cultivated and wild	1	1	2	2
54K	Cultivated	Omo	Cultivated and wild	2	2	1	1
55k	Cultivated	Omo	Cultivated and wild	2	2	1	1
56k	Cultivated	Omo	Cultivated and wild	2	2	1	1
60k	Wild	Keffa	Wild	2	2	1	1
61k	Cultivated	Omo	Cultivated and wild	1	1	Uncluster	Unclustered
63k	Cultivated	Omo	Cultivated and wild	1	1	2	2
6s	Wild	Sheka	Wild	2	2	1	1
