# Supplementary material for: Accumulation of mutations in genes associated with sexual reproduction contributed to the domestication of a vegetatively propagated staple crop, enset
Source: Hortic Res. 2020 Nov 1;7:185. doi: 10.1038/s41438-020-00409-7 (PMC7603512; doi:10.1038/s41438-020-00409-7)
Supplement: Supplementary file 17 — Supplementary Table 7 [file 41438_2020_409_MOESM17_ESM.pdf]

Supplementary Table 7: Summary of cultivated and wild enset accessions sampled from six regions in South and South Western Ethiopia.

Region	Sub-region	Number of households	Number of samples		Total samples
			cultivated	wild	
Dawro	Loma	7	16	1	17
	Mareka	5	7	2	9
Guragie	Cheha	5	7	0	7
	Gumer	8	22	0	22
Holeta	Holeta	1	11	0	11
Keffa	Chena	7	20	4	24
	Decha	8	25	13	38
Omo	South Ari	10	36	6	42
Sheka	Benji		0	4	4
	Tepi		0	8	8
Sidama	Shebedino	6	19	0	19
	Korcchie	7	29	0	29
Total		64	192	38	230
