# Supplementary material for: Accumulation of mutations in genes associated with sexual reproduction contributed to the domestication of a vegetatively propagated staple crop, enset
Source: Hortic Res. 2020 Nov 1;7:185. doi: 10.1038/s41438-020-00409-7 (PMC7603512; doi:10.1038/s41438-020-00409-7)
Supplement: Supplementary file 18 — Supplementary Table 8 [file 41438_2020_409_MOESM18_ESM.pdf]

Supplementary Table 8: Accession name, area of collection, longitude and latitude information of samples used for AFLP analysis

ID	Accession name	Regions	Subregion	Domestic/ Wild	Longitude	Latitude	Inferred Cluster (based on population STRCUTRE analysis, *K*=3)
14d	Shededini	Dawro	Loma	Domestic	299758.4	779793.6	3
15d	Arkia white	Dawro	Loma	Domestic	299758.4	779793.6	1
16d	Boza	Dawro	Loma	Domestic	299758.4	779793.6	2
19d	Shededini	Dawro	Loma	Domestic	299758.4	779793.6	1
1d	Mazia	Dawro	Loma	Domestic	299758.4	779793.6	1
24d	Chiche	Dawro	Loma	Domestic	299758.4	779793.6	1
28d	Boduntuwa	Dawro	Loma	Domestic	299758.4	779793.6	1
29d	Gena	Dawro	Loma	Domestic	299758.4	779793.6	1
2d	mazia white	Dawro	Loma	Domestic	299758.4	779793.6	2
33d	bella	Dawro	Loma	Domestic	299758.4	779793.6	1
34d	Shesho	Dawro	Loma	Domestic	299758.4	779793.6	1
3d	Shasho	Dawro	Loma	Domestic	299758.4	779793.6	2
6d	Amia	Dawro	Loma	Domestic	299758.4	779793.6	1
8d	Boza	Dawro	Loma	Domestic	299758.4	779793.6	2
21d	Mazia black	Dawro	Loma	Domestic	299758.4	779793.6	1
27d	Tuzuma	Dawro	Loma	Domestic	299758.4	779793.6	Not clustered
7d	wild	Dawro	Loma	Wild	299758.4	779793.6	3
10d	mazia white	Dawro	Mareka	Domestic	299370.6	780027.6	2
20d	lonchingie	Dawro	Mareka	Domestic	299370.6	780027.6	1
25d	Agina white	Dawro	Mareka	Domestic	299370.6	780027.6	2
30d	Tiella	Dawro	Mareka	Domestic	299370.6	780027.6	3
32d	yeka	Dawro	Mareka	Domestic	299370.6	780027.6	1
35d	Amia boduntuwa	Dawro	Mareka	Domestic	299370.6	780027.6	1
9d	Dika	Dawro	Mareka	Domestic	299370.6	780027.6	3
17d	wild	Dawro	Mareka	Wild	299370.6	780027.6	3
4d	wild	Dawro	Mareka	Wild	299370.6	780027.6	3
10g	Kimnar	Guragie	Cheha	Domestic	384479.4	895562.4	2
11g	Kinkie	Guragie	Cheha	Domestic	384479.4	895562.4	2
12g	Engida	Guragie	Cheha	Domestic	384479.4	895562.4	1
22g	Konchwa	Guragie	Cheha	Domestic	384479.4	895562.4	2
3g	lamot	Guragie	Cheha	Domestic	384479.4	895562.4	2
4g	Gariye	Guragie	cheha	Domestic	384479.4	895562.4	2
5g	Euret	Guragie	cheha	Domestic	384479.4	895562.4	2
13g	Embiwa	Guragie	Gumer	Domestic	395597.4	873656.7	2
14g	mcp	Guragie	Gumer	Domestic	395597.4	873656.7	2
15g	gariye	Guragie	Gumer	Domestic	395597.4	873656.7	2
16g	yesra chinchie	Guragie	Gumer	Domestic	395597.4	873656.7	2
17g	Mintigire	Guragie	Gumer	Domestic	395597.4	873656.7	2

18g	benazia	Guragie	Gumer	Domestic	395597.4	873656.7	2
19g	Agadie	Guragie	Gumer	Domestic	395597.4	873656.7	2
1g	Ayshro	Guragie	Gumer	Domestic	395597.4	873656.7	2
20g	Derie	Guragie	Gumer	Domestic	395597.4	873656.7	2
21g	Ankafiya	Guragie	Gumer	Domestic	395597.4	873656.7	3
23g	Astara	Guragie	Gumer	Domestic	395597.4	873656.7	2
24g	Shertie	Guragie	Gumer	Domestic	395597.4	873656.7	2
25g	Amerat	Guragie	Gumer	Domestic	395597.4	873656.7	2
26g	Kembat	Guragie	Gumer	Domestic	395597.4	873656.7	2
27g	Separa	Guragie	Gumer	Domestic	395597.4	873656.7	2
28g	Kibnar	Guragie	Gumer	Domestic	395597.4	873656.7	2
29g	Bededit	Guragie	Gumer	Domestic	395597.4	873656.7	2
2g	Gimbo	Guragie	Gumer	Domestic	395597.4	873656.7	2
6g	yirgie	Guragie	Gumer	Domestic	395597.4	873656.7	2
7g	Tegeded	Guragie	Gumer	Domestic	395597.4	873656.7	2
8g	Denkinet	Guragie	Gumer	Domestic	395597.4	873656.7	2
9g	Yestra Chenech	Guragie	Gumer	Domestic	395597.4	873656.7	2
10HO	gewada	Holeta	Holeta	Domestic	445592.7	1002222.9	2
11HO	mesena	Holeta	Holeta	Domestic	445592.7	1002222.9	2
1HO	Abel	Holeta	Holeta	Domestic	445592.7	1002222.9	2
2HO	Adelcho	Holeta	Holeta	Domestic	445592.7	1002222.9	1
3HO	Mazia	Holeta	Holeta	Domestic	445592.7	1002222.9	2
4HO	Yambulie	Holeta	Holeta	Domestic	445592.7	1002222.9	1
5HO	kelisa	Holeta	Holeta	Domestic	445592.7	1002222.9	2
6HO	abalgove	Holeta	Holeta	Domestic	445592.7	1002222.9	2
7HO	zerta	Holeta	Holeta	Domestic	445592.7	1002222.9	2
8HO	endale	Holeta	Holeta	Domestic	445592.7	1002222.9	2
9HO	Arkia	Holeta	Holeta	Domestic	445592.7	1002222.9	2
58K	Bajo	Keffa	Cheha	Domestic	384479.4	895562.4	1
2k	Unknown	Keffa	Chena	Domestic	384479.4	895562.4	1
22k	Hichawi	Keffa	Chena	Domestic	384479.4	895562.4	1
21k	R.Boso	Keffa	Chena	Domestic	384479.4	895562.4	1
1k	Agenas	Keffa	Chena	Domestic	384479.4	895562.4	1
20k	boso white	Keffa	Chena	Domestic	384479.4	895562.4	1
25k	Gomijo	Keffa	Chena	Domestic	384479.4	895562.4	1
26k	Kupro	Keffa	Chena	Domestic	384479.4	895562.4	3
27k	Chero	Keffa	Chena	Domestic	384479.4	895562.4	1
29k	Kalo	Keffa	Chena	Domestic	384479.4	895562.4	1
32k	Kocha Gashew	Keffa	Chena	Domestic	384479.4	895562.4	1
33k	Bedo	Keffa	Chena	Domestic	384479.4	895562.4	1
35k	Ageni	Keffa	Chena	Domestic	384479.4	895562.4	3
38k	Areko white (3)	Keffa	Chena	Domestic	384479.4	895562.4	1
40k	Kitanafi	Keffa	Chena	Domestic	384479.4	895562.4	1
47k	Yodiafo	Keffa	Chena	Domestic	384479.4	895562.4	1
5k	machedemi	Keffa	Chena	Domestic	384479.4	895562.4	2

62K	chora	Keffa	Chena	Domestic	384479.4	895562.4	1
63K	Areka white	Keffa	Chena	Domestic	384479.4	895562.4	1
8k	Adelbecho	Keffa	Chena	Domestic	384479.4	895562.4	2
6k	wild	Keffa	Chena	Wild	384479.4	895562.4	3
7k	wild	Keffa	Chena	Wild	384479.4	895562.4	3
9k	wild	Keffa	Chena	Wild	384479.4	895562.4	3
30k	wild	Keffa	chena	Wild	384479.4	895562.4	3
12k	mecho	Keffa	Decha	Domestic	805967.8	788040.3	2
14k	shimo	Keffa	Decha	Domestic	805967.8	788040.3	1
16k	Bango	Keffa	Decha	Domestic	805967.8	788040.3	1
17k	kiekero	Keffa	Decha	Domestic	805967.8	788040.3	1
18k	Gayo	Keffa	Decha	Domestic	805967.8	788040.3	3
19k	Betecho	Keffa	Decha	Domestic	805967.8	788040.3	1
28k	Bekellbecho	Keffa	Decha	Domestic	805967.8	788040.3	1
31k	shelako	Keffa	Decha	Domestic	805967.8	788040.3	1
34k	Bejo	Keffa	Decha	Domestic	805967.8	788040.3	1
36k	Bena	Keffa	Decha	Domestic	805967.8	788040.3	Not clustered
39k	Terredo	Keffa	Decha	Domestic	805967.8	788040.3	1
3k	mzia white	Keffa	Decha	Domestic	805967.8	788040.3	2
44k	Kupo	Keffa	Decha	Domestic	805967.8	788040.3	1
45k	Gonjibecho	Keffa	Decha	Domestic	805967.8	788040.3	1
46k	Yako	Keffa	Decha	Domestic	805967.8	788040.3	1
48k	Areko white	Keffa	Decha	Domestic	805967.8	788040.3	1
49K	Tayo	Keffa	Decha	Domestic	805967.8	788040.3	1
4k	Gena	Keffa	Decha	Domestic	805967.8	788040.3	1
51K	Cherkayo	Keffa	Decha	Domestic	805967.8	788040.3	1
53K	Rochtato	Keffa	Decha	Domestic	805967.8	788040.3	1
54K	Arkabo	Keffa	Decha	Domestic	805967.8	788040.3	1
55K	Etro	Keffa	Decha	Domestic	805967.8	788040.3	1
56K	Tuto	Keffa	Decha	Domestic	805967.8	788040.3	1
59K	bombo	Keffa	Decha	Domestic	805967.8	788040.3	1
61K	omo	Keffa	Decha	Domestic	805967.8	788040.3	1
10k	wild	Keffa	Decha	Wild	805967.8	788040.3	1
11k	wild	Keffa	Decha	Wild	805967.8	788040.3	3
13k	wild	Keffa	Decha	Wild	805967.8	788040.3	3
15k	wild	Keffa	Decha	Wild	805967.8	788040.3	1
23k	wild	Keffa	Decha	Wild	805967.8	788040.3	1
24k	wild	Keffa	Decha	Wild	805967.8	788040.3	3
37k	wild	Keffa	Decha	Wild	805967.8	788040.3	3
41k	wild	Keffa	Decha	Wild	805967.8	788040.3	2
42k	wild	Keffa	Decha	Wild	805967.8	788040.3	3
43k	wild	Keffa	Decha	Wild	805967.8	788040.3	3
50K	wild	Keffa	Decha	Wild	805967.8	788040.3	3
52K	wild	Keffa	Decha	Wild	805967.8	788040.3	3

60K	wild	Keffa	Decha	Wild	805967.8	788040.3	3
10m	Mala	Omo	S.Ari	Domestic	260890.1	260890.1	2
11m	Tsoka	Omo	S.Ari	Domestic	260890.1	260890.1	2
12m	Dema	Omo	S.Ari	Domestic	260890.1	260890.1	2
14m	Yefereng kocho	Omo	S.Ari	Domestic	260890.1	260890.1	2
15m	Tsela	Omo	S.Ari	Domestic	260890.1	260890.1	2
16m	Arfa	Omo	S.Ari	Domestic	260890.1	260890.1	1
1m	Gedes	Omo	S.Ari	Domestic	260890.1	260890.1	1
28m	Delio	Omo	S.Ari	Domestic	260890.1	260890.1	1
2m	yintie	Omo	S.Ari	Domestic	260890.1	260890.1	1
30m	Molet	Omo	S.Ari	Domestic	260890.1	260890.1	2
31m	Kerta	Omo	S.Ari	Domestic	260890.1	260890.1	2
32m	Shuferek	Omo	S.Ari	Domestic	260890.1	260890.1	1
33m	Tsami	Omo	S.Ari	Domestic	260890.1	260890.1	2
34m	Salta	Omo	S.Ari	Domestic	260890.1	260890.1	1
36m	Zinka	Omo	S.Ari	Domestic	260890.1	260890.1	1
37m	Gena	Omo	S.Ari	Domestic	260890.1	260890.1	2
38m	Gena	Omo	S.Ari	Domestic	260890.1	260890.1	2
3m	gufrek	Omo	S.Ari	Domestic	260890.1	260890.1	3
40m	Bubumi	Omo	S.Ari	Domestic	260890.1	260890.1	1
41m	Berga	Omo	S.Ari	Domestic	260890.1	260890.1	1
42m	Monet	Omo	S.Ari	Domestic	260890.1	260890.1	1
44m	Kalchakim	Omo	S.Ari	Domestic	260890.1	260890.1	1
45m	Kekish	Omo	S.Ari	Domestic	260890.1	260890.1	1
46m	Basmatoch	Omo	S.Ari	Domestic	260890.1	260890.1	1
47m	Tsofrak	Omo	S.Ari	Domestic	260890.1	260890.1	1
48m	Gaya	Omo	S.Ari	Domestic	260890.1	260890.1	1
49m	Dekay	Omo	S.Ari	Domestic	260890.1	260890.1	1
4m	shoka	Omo	S.Ari	Domestic	260890.1	260890.1	1
50m	Arbi	Omo	S.Ari	Domestic	260890.1	260890.1	2
51m	Weshemach	Omo	S.Ari	Domestic	260890.1	260890.1	1
52m	Siki	Omo	S.Ari	Domestic	260890.1	260890.1	1
5m	Ziregna	Omo	S.Ari	Domestic	260890.1	260890.1	1
6m	Bukuma	Omo	S.Ari	Domestic	260890.1	260890.1	2
8m	Goliot	Omo	S.Ari	Domestic	260890.1	260890.1	1
9m	Goliot	Omo	S.Ari	Domestic	260890.1	260890.1	Not clustered
19m	Kaka	Omo	S.Ari	Domestic	260890.1	260890.1	2
13m	wild	Omo	S.Ari	Wild	260890.1	260890.1	3
17m	Wild	Omo	S.Ari	Wild	260890.1	260890.1	3
23m	wild	Omo	S.Ari	Wild	260890.1	260890.1	3
29m	wild	Omo	S.Ari	Wild	260890.1	260890.1	2
39m	wild	Omo	S.Ari	Wild	260890.1	260890.1	3
43m	wild	Omo	S.Ari	Wild	260890.1	260890.1	2
1B	wild	Sheka	Benchi Manji	Wild	197989	774627.3	3

2B	wild	Sheka	Benchi Manji	Wild	197989	774627.3	3
3B	wild	Sheka	Benchi Manji	Wild	197989	774627.3	3
4B	wild	Sheka	Benchi Manji	Wild	197989	774627.3	3
3S	wild	Sheka	Tepi	Wild	766859.5	802096.2	3
1S	wild	Sheka	Tepi	Wild	766859.5	802096.2	3
2S	wild	Sheka	Tepi	Wild	766859.5	802096.2	3
4S	wild	Sheka	Tepi	Wild	766859.5	802096.2	3
6S	wild	Sheka	Tepi	Wild	766859.5	802096.2	3
7S	wild	Sheka	Tepi	Wild	766859.5	802096.2	3
8S	wild	Sheka	Tepi	Wild	766859.5	802096.2	3
9S	wild	Sheka	Tepi	Wild	766859.5	802096.2	3
11h	Gedime	Sidama	Gorchie	Domestic	435638.5	759532.2	1
12h	Midasho	Sidama	Gorchie	Domestic	435638.5	759532.2	2
13h	Gechero	Sidama	Gorchie	Domestic	435638.5	759532.2	2
16h	kulie	Sidama	Gorchie	Domestic	435638.5	759532.2	2
20h	terora	Sidama	Gorchie	Domestic	435638.5	759532.2	2
22h	gulumo	Sidama	Gorchie	Domestic	435638.5	759532.2	2
23h	ganticho	Sidama	Gorchie	Domestic	435638.5	759532.2	2
25h	askala	Sidama	Gorchie	Domestic	435638.5	759532.2	2
27h	ado (3)	Sidama	Gorchie	Domestic	435638.5	759532.2	2
28h	Banja	Sidama	Gorchie	Domestic	435638.5	759532.2	2
29h	dergicha	Sidama	Gorchie	Domestic	435638.5	759532.2	2
31h	hekichie	Sidama	Gorchie	Domestic	435638.5	759532.2	2
32h	gamachala	Sidama	Gorchie	Domestic	435638.5	759532.2	2
34h	gademi	Sidama	Gorchie	Domestic	435638.5	759532.2	2
37h	harbigoncho	Sidama	Gorchie	Domestic	435638.5	759532.2	2
45h	balicho	Sidama	Gorchie	Domestic	435638.5	759532.2	2
46h	kanbaticho	Sidama	Gorchie	Domestic	435638.5	759532.2	1
47h	bufaro	Sidama	Gorchie	Domestic	435638.5	759532.2	2
5h	Kiticho	Sidama	Gorchie	Domestic	435638.5	759532.2	2
10h	Birbo	Sidama	Shebedino	Domestic	452258.7	759273	2
14h	Adara	Sidama	Shebedino	Domestic	452258.7	759273	2
15h	Checho (3)	Sidama	Shebedino	Domestic	452258.7	759273	2
17h	gosalo	Sidama	Shebedino	Domestic	452258.7	759273	2
18h	Checho (2)	Sidama	Shebedino	Domestic	452258.7	759273	2
1h	Goloma	Sidama	Shebedino	Domestic	452258.7	759273	2
24h	waniwsa	Sidama	Shebedino	Domestic	452258.7	759273	2
26h	borbodicho	Sidama	Shebedino	Domestic	452258.7	759273	2
2h	Siraro	Sidama	Shebedino	Domestic	452258.7	759273	2
30h	ewishi	Sidama	Shebedino	Domestic	452258.7	759273	1
33h	sedso	Sidama	Shebedino	Domestic	452258.7	759273	2
35h	bowie	Sidama	Shebedino	Domestic	452258.7	759273	2
36h	ado(1)	Sidama	Shebedino	Domestic	452258.7	759273	2

38h	dawramo	Sidama	Shebedino	Domestic	452258.7	759273	2
39h	gena	Sidama	Shebedino	Domestic	452258.7	759273	2
3h	Alaticho	Sidama	Shebedino	Domestic	452258.7	759273	2
40h	sedra	Sidama	Shebedino	Domestic	452258.7	759273	2
41h	botetie	Sidama	Shebedino	Domestic	452258.7	759273	1
42h	Checho (1)	Sidama	Shebedino	Domestic	452258.7	759273	1
43h	ado (2)	Sidama	Shebedino	Domestic	452258.7	759273	2
44h	haho	Sidama	Shebedino	Domestic	452258.7	759273	2
48h	kanda	Sidama	Shebedino	Domestic	452258.7	759273	2
49h	Munderaro (2)	Sidama	Shebedino	Domestic	452258.7	759273	2
4h	Bira	Sidama	Shebedino	Domestic	452258.7	759273	2
50h	Munderaro (3)	Sidama	Shebedino	Domestic	452258.7	759273	2
6h	Agena	Sidama	Shebedino	Domestic	452258.7	759273	2
7h	Hawie	Sidama	Shebedino	Domestic	452258.7	759273	2
8h	Demela	Sidama	Shebedino	Domestic	452258.7	759273	1
9h	Munderaro	Sidama	Shebedino	Domestic	452258.7	759273	2
