# Supplementary material for: Accumulation of mutations in genes associated with sexual reproduction contributed to the domestication of a vegetatively propagated staple crop, enset
Source: Hortic Res. 2020 Nov 1;7:185. doi: 10.1038/s41438-020-00409-7 (PMC7603512; doi:10.1038/s41438-020-00409-7)
Supplement: Supplementary file 19 — Supplementary Table 9 [file 41438_2020_409_MOESM19_ESM.pdf]

**Supplementary Table 9:** Sequences of oligonucleotide used for AFLP and GBS. Selective bases in the primers used during the pre-selective and selective amplifications are highlighted in bold. Unique GBS barcode bases are represented as X.

| Oligo name | Function | Sequence |
|---|---|---|
| *Msp*I adaptor | Reverse Adaptor | CGCTCAGGACTCAT |
| *Msp*I adaptor | Forward Adaptor | GACGATGAGTCCTGAG |
| *Eco*RI adaptor | Reverse Adaptor | AATTGGTACGCAGTCTAC |
| *Eco*RI adaptor | Forward Adaptor | CTCGTAGACTGCGTACC |
| Pre- EcoRI | Pre-selective primer | GACTGCGTACCAATTC**A** |
| Pre- *Msp*I | Pre-selective primer | GATGAGTCCTGAGCGG**C** |
| *Eco*RI Selective Primer | Selective primer | GACTGCGTACCAATTC**ACG** |
| *Msp*I Selective Primer | Selective primer | GATGAGTCCTGAGCGG**CAA** |
| *Msp*I GBS barcoded adaptor | Reverse Adaptor | CGXXXXAGATCGGAAGAGCGTCGTGTAGGGAAAGAGTGT |
| *Msp*I GBS barcoded adaptor | Forward Adaptor | ACACTCTTTCCCTACACGACGCTCTTCCGATCTXXXX |
| *Eco*RI GBS Y adaptor | Reverse Adaptor | CGAGATCGGAAGAGCGGTTCAGCAGGAATGCCGAG |
| *Eco*RI GBS Y adaptor | Forward Adaptor | CTCGGCATTCCTGCTGAACCGCTCTTCCGATCT |
| *Msp*I GBS primer | Sequencing library primers | AATGATACGGCGACCACCGAGATCTACACTCTTTCCCTACACGACGCTCTTCCGATCT |
| *Eco*RI GBS primer | | CAAGCAGAAGACGGCATACGAGATCGGTCTCGGCATTCCTGCTGAACCGCTCTTCCGATCT |
